# Dynamics and Interplay of the Binding Pockets in the Glycine Receptor

**DOI:** 10.1021/acs.jcim.5c00340

**Published:** 2025-07-31

**Authors:** Guangpeng Xue, Jacob Adam Clark, Cambrin Kemble-Diaz, Alessandro Crnjar, Carla Molteni

**Affiliations:** Physics Department, 4616 King’s College London, Strand, London WC2R 2LS, U.K.

## Abstract

The glycine receptor, a pentameric ligand-gated ion channel, plays a vital role in inhibitory neurotransmission, reflexes, and neuronal excitability. It is crucial to maintaining the balance and responsiveness of the nervous system to sensory input. The binding of ligands, in this case, glycines, in the extracellular domain (ECD) of the receptor initiates a series of conformational rearrangements that culminate in the opening of the ion channel in the transmembrane domain. There are five binding sites for orthosteric ligands at the interface among the five subunits of the receptor. Experiments suggest that two or three bound glycines are sufficient to activate the receptor and that the occupancy of binding sites affects (un)­binding rates. Here, we evaluated the dynamics and interplay of empty and occupied binding pockets and their potential cooperativity. We investigated ECD models for the glycine receptor, built from cryo-EM data, by performing molecular dynamics simulations for different combinations of ligand occupancies. We highlighted the role of glycine in contracting the binding site, optimizing the water content to the amount necessary to mediate crucial interactions and dragging Loop C to cover the pocket. Each subunit participates in two adjacent binding pockets acting, in turn, as the principal and complementary subunit, with structures such as Loop B and Loop F being directly connected. This suggests a combination of push–pull mechanisms mediated by ligands in a potentially frustrated system, which may favor specific occupancy patterns or alternation of binding sites with different levels of contraction.

## Graphical abstract

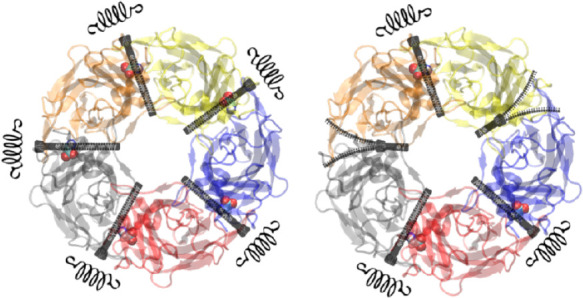

## Introduction

Glycine receptors (GlyRs) are pentameric ligand-gated chloride channels (pLGICs) that mediate inhibitory neurotransmission. They are abundantly expressed throughout the central nervous system and are found primarily in the spinal cord, brain stem, and retina. In the spinal cord, postsynaptic GlyRs are important in regulating motor reflexes and controlling movement. Presynaptic GlyRs are found at glutamatergic and glycinergic synapses and play a pivotal role in modulating neurotransmitter release.^1–6^

The GlyRs belong to the superfamily of Cys-loop pLGICs. In vertebrates, this superfamily of pLGICs can be classified into two categories: cation-selective receptors, which include 5-HT_3_ serotonin receptors and nicotinic acetylcholine receptors (nAChRs), and anion-selective receptors, which include GABA and glycine receptors.^7^ Structurally, as for other pLGICs, GlyR is a pentameric assembly of five homologous or heterologous subunits. To date, there are four known α subunits (α1−α4) and one β subunit. From biochemical analyses, GlyR heteropentamers were predicted with either three α and two β subunits or with two α and three β subunits.^8–10^ However, recent structural studies revealed that heteromeric GlyRs have an unexpected 4:1 α:β subunit stoichiometry.^11–14^ Homomeric receptors are believed to be more prevalent in young animals but are usually replaced with αβ heteromers in adults. However, there is evidence suggesting the continued presence of homomeric glycine receptors in adult animals.^15^

Each GlyR subunit is composed of three domains: an extracellular domain (ECD), a transmembrane domain (TMD), and an intracellular domain (ICD). The ECD is mostly made of β-sheets and hosts five orthosteric ligand-binding sites at the interface between subunits. The TMD is made of four α-helices (M1–M4) embedded in the cell membrane. The M1 helix connects to and anchors the ECD, which is outside the cell membrane, to the TMD, while the M2 helix lines the channel. The M3 and M4 helices connect to the ICD, with M4 at the interface with the membrane.^16^ Figure 1 illustrates the entire typical protein structure of a pLGIC, including the three domains, the subunits, the membrane, and the bound agonists. It is believed that two or three agonists binding to the ECD pockets are sufficient to activate the receptor, triggering the ion channels to open through a series of conformational changes, including hard-to-capture “flip” states.^17,18^

**Figure 1:**
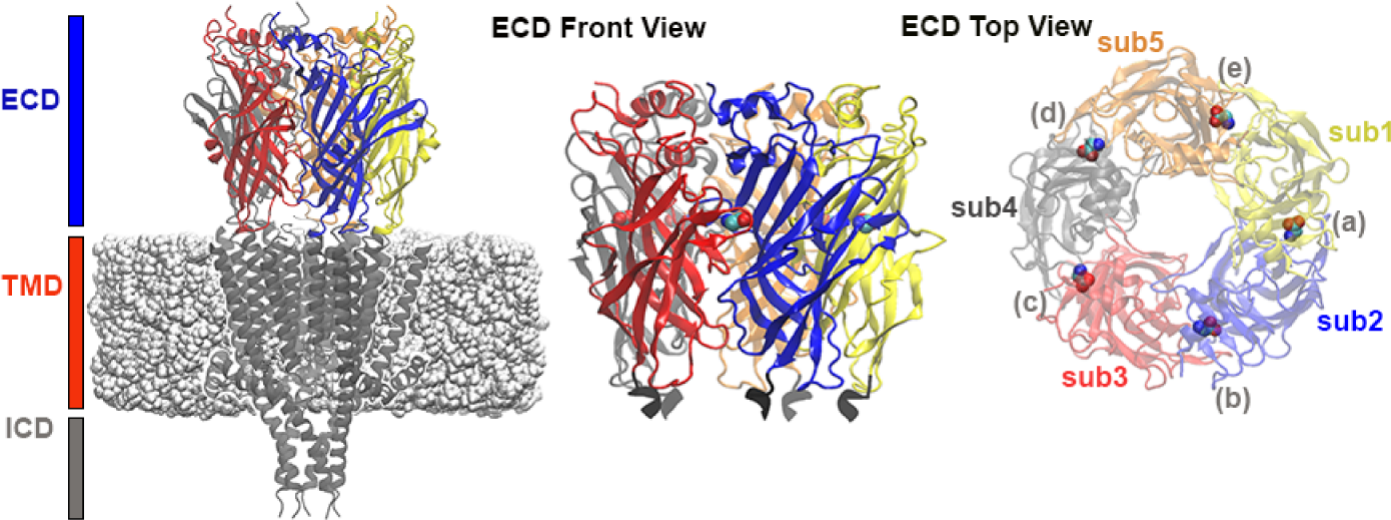
On the left: general structure of a pLGIC embedded in the membrane. In the middle: front view of the ECD model for the zebrafish homopentameric α_1_ GlyR (PDB entry: 6PM5 ^17^), with subunits shown in different colors; the restrained residues at the interface with the TMD are represented as dark gray ribbons. On the right: top view of the GlyR ECD, with the orthosteric binding pockets (labeled from (a) to (e)) at the interface between adjacent subunits (labeled from sub1 to sub5), with bound glycine agonists.

Each ligand-binding site in the ECD is made up of two twisted β-sheets. Three β-strands make up the outer binding site, while seven β-strands make up the inner (vestibule-facing) binding site.^19^ Seven loops (labeled A-C and Loop 2 in the principal subunit and D-F in the complementary subunit) border the ligand-binding site, as shown in Figure 2i.^20–26^

**Figure 2:**
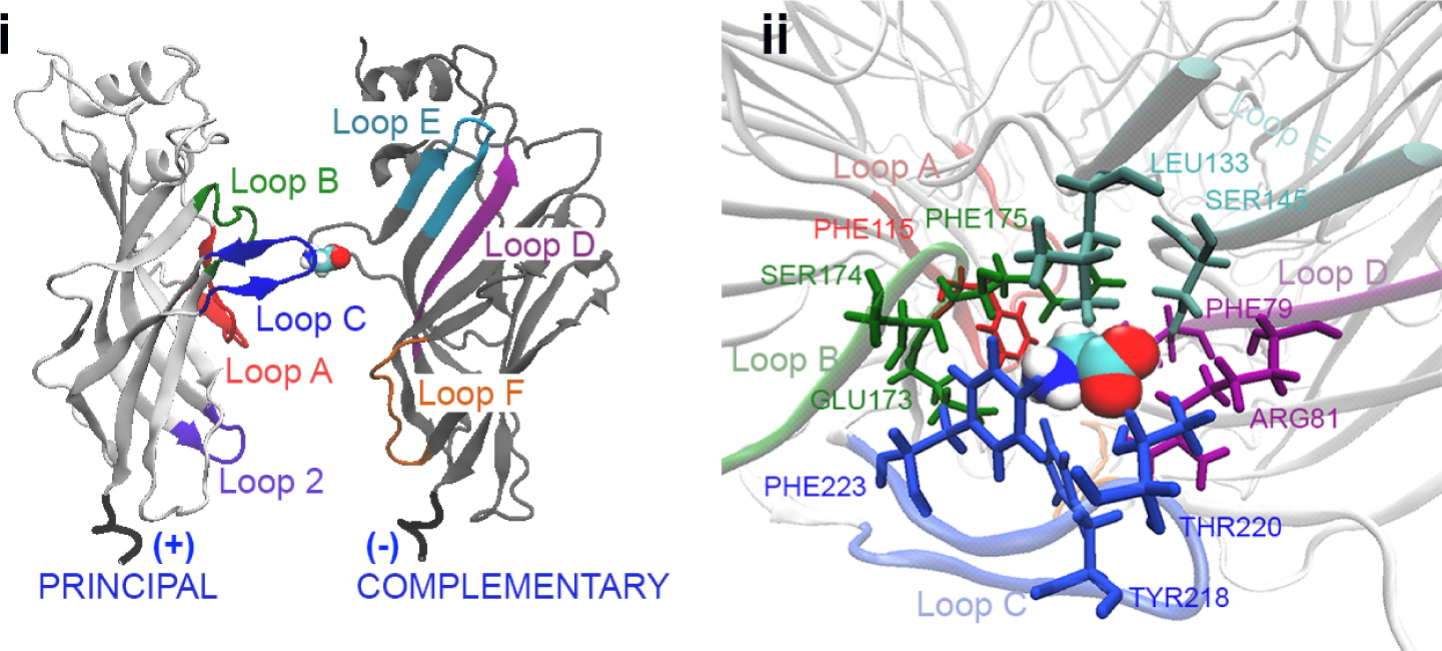
(i) Principal and complementary subunits of the GlyR ECD model (PDB entry: 6PM5 ^17^) with the loops forming the binding site highlighted. (ii) Main residues of the glycine binding pocket colored according to the loop to which they belong; glycine is shown in van der Waals style.

The ligands that activate pLGICs are classified as full agonists and partial agonists according to their efficacy in triggering ion channel opening. For example, in the zebrafish homomeric α1 GlyR, glycine is the full agonist with EC_50_ = 190 ± 20 μM,^17^ but there are several partial agonists: β-alanine is a structural analogue of glycine with EC_50_ = 0.97 mM;^27^ taurine is less efficient with EC_50_ = 1.05 ± 0.08 mM;^17^ and γ-aminobutyric acid (GABA) is a very weak agonist with EC_50_ = 28.4 ± 0.9 mM.^17^

Little information is known about the cooperation and interaction between subunits upon (un)­binding. Experimental studies, based on single-channel recording on the rat recombinant α1GlyR,^15^ showed that gating efficacy increased with the level of ligation, reaching a maximum with three bound ligands, although any number of glycines from one to five could open the ion channel. The dissociation rate constants from the channel closed state increased when the number of bound molecules increased from one to three, indicating negatively cooperative interactions between binding sites. However, the association rate constants appeared to show positively cooperative interactions, being slow for the first binding but increasing for subsequent bindings. Mutagenesis experiments on the human heteromeric α_1_β (4:1) GlyR showed that two binding sites at α1−α1 interfaces were sufficient for activation and necessary for high efficacy.^11^ Coarse-grained free energy simulations, where a pathway between closed, open, and desensitized states was generated by targeted molecular dynamics in the homomeric α1 GlyR, concluded that the activation barrier decreased with an increasing number of bound glycines, reaching a threshold value for efficient activation with three nonconsecutive or more occupied binding sites. Moreover, they showed that the desensitization barrier initially decreased but increased upon more glycine binding; seven different binding patterns obtained by docking were tested, and the study was complemented by mutagenesis electrophysiology experiments guided by computational mutation scans.^28^ Molecular dynamics (MD) simulations on the bacterial homomeric pLGIC GLIC, allosterically bound to the anesthetic propofol in the TMD, showed that symmetry breaking by different propofol occupancies promoted changes in channel conformation.^29^ The effects of different numbers of trans–cis proline switches, proposed as mediators for channel gating, at the interface between the ECD and TMD were also computationally explored in a model of the homomeric 5-HT_3A_ receptor.^30^ Studies of the GlyR fluorescently labeled at the ECD-TMD interface by voltage-clamp fluorometry were able to capture intermediate states during activation.^31^ The authors speculate that combinations of agonist and antagonist binding, partial agonist binding, or partial glycine occupation of the binding sites stabilize an intermediate closed state preceding gating, which is characterized by local asymmetry in the ECD.

Elucidating the interactions between bound and unbound pockets is key to understanding the potential cooperative mechanisms underlying channel opening. With this goal in mind, we built four GlyR ECD models, based on cryo-EM structural data, with different patterns of ligand occupancy, as sketched in Figure 3. We employed MD, which has proven to be very useful in the investigations of pLGICs as well as other ion channels,^32–39^ to perform all-atom simulations of these systems. By evaluating the differences between the pocket dynamics, we probed how the five subunits interact at the ECD level and how neighboring pockets may cooperate. Atomic-level insights into the dynamics and interplay of the binding pockets are important to better understand how these receptors function and malfunction, and may ultimately contribute to opening new avenues for drug development.^40,41^

**Figure 3:**
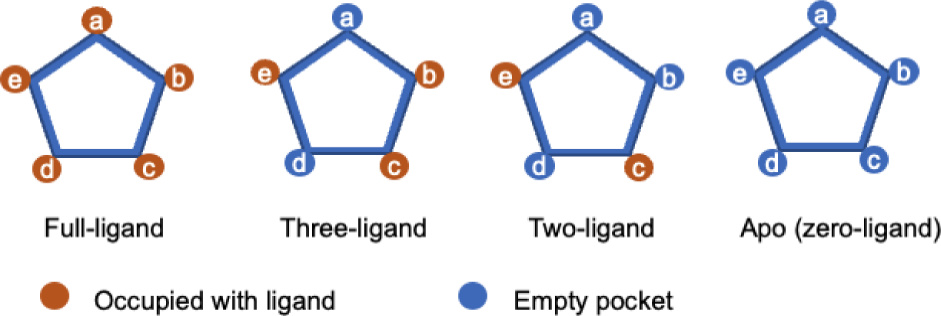
Schemes of the four ECD models of GlyR with different numbers of bound ligands. The full-ligand model was constructed based on the PDB entry: 6PM5;^17^ the three- and two-ligand models were obtained by unbinding two or three ligands from the full-ligand model; the Apo (zero-ligand) model was constructed based on the PDB entry: 6PXD.^17^

## Methods

We built a model of the extracellular domain from the cryo-EM experimental structure of a homomeric zebrafish α1 glycine receptor in a desensitized state with a resolution of 3.10 Å (PDB entry: 6PM5 ^17^). Glycine is bound in all five equivalent pockets. This structure was released together with other structures of comparable resolution in different functional states and with different bound ligands, which makes them suitable as starting points for atomistic simulations on various aspects of the activation mechanisms. The ECD of the open and that of the more stable desensitized structure of the GlyR are very similar, with differences in the two states confined to the transmembrane domain;^11,17^ hence, we selected the ECD of the desensitized state. For comparison, we also built an ECD model of the Apo (closed, no bound ligands) structure, based on PDB entry 6PXD, at a resolution of 2.90 Å.^17^

The opening of the channel requires interactions of the ECD and the TMD domains, which occur at timescales inaccessible to all-atom MD simulations. Consistently with other studies of pLGICs focused on binding,^42–45^ we limited our analysis to the ECD, which contains the orthosteric binding pockets of interest. While this is a limitation, it allowed for more efficient simulations without affecting the regions of the binding pockets within the timescale considered here. The ECD model contains 218 residues per subunit, including the whole ECD structure and one residue of the pre-M1 helix; the last five residues of each subunit were restrained during the MD simulations to mimic the anchor effect of the TMD, as in previous ECD simulations.^42,44,45^ Hydrogen atoms were added using the web server H++ at neutral pH.^46^ Crucial disulfide bonds (forming the characteristic Cys-loop of the pLGIC superfamily GlyR belongs to) were identified and modeled.^47,48^ The protein was solvated with a 15 Å buffer of TIP3P water in an orthorhombic cell, with 0.15 M Na^+^ and Cl^–^ ions included to mimic physiological conditions, and the whole system was neutralized by balancing the number of ions. The final structure consists of 123 299 atoms, including protein, water, ions, and glycine ligands in zwitterionic form, in an initial cell of dimensions 110 Å × 110 Å × 110 Å. The ff14SB Amber force field^49^ was used. All MD simulations were performed with NAMD 2.14 ^50,51^ with a time step of 2 fs and the SHAKE algorithm to restrict the lengths of the bonds involving hydrogen atoms. The Particle Mesh Ewald algorithm was used for electrostatic interactions, and a cutoff of 10 Å, accompanied by a switching function, was employed for all nonbonded interactions.

The zwitterionic form of glycine was parameterized using the GAFF force field.^52^ For consistency with the Amber force field, the ligand partial charges were calculated at the Hartree–Fock level with a 6-31G* basis set and a RESP fitting procedure on a geometry-optimized structure obtained at the density functional theory level with the B3LYP exchange and correlation functional and the same basis set with Gaussian 09.^53^ As the geometry optimization was carried out in vacuo, to avoid proton transfer, the bonds containing hydrogen of the ammonium moiety were constrained.

The system was subjected to minimization, thermalization, and equilibration procedures, where harmonic restraints on the protein and ligands were progressively removed, except for those on the backbone of the last five residues of each subunit (residues 236 to 240 according to the PDB numbering), which were kept at 1 kcal/(mol Å^2^). This was followed by a 1000 ns production MD in the NPT ensemble at 310 K and 1 atm. The temperature was controlled by a Langevin thermostat with a coupling coefficient of 1 ps^–1^. The pressure was controlled by a Langevin piston barostat, with an oscillation period of 200 fs and a damping timescale of 100 fs.

Following the completion of the MD production run for the original model, well-tempered metadynamics was applied using PLUMED^54,55^ to eject two or three ligands according to the scheme in Figure 3: this procedure produced the two-ligand and three-ligand models, respectively. The collective variables for the metadynamics were the distance between the center of mass (COM) of the binding residues and that of the ligand and the ligand solvation.

The latter was defined as $\underset{i}{\sum }\underset{j}{\sum }\frac{1-{\left(\frac{d_{ij}}{d_{0}}\right)}^{6}}{1-{\left(\frac{d_{ij}}{d_{0}}\right)}^{10}}$ where *d*_*ij*_ is the distance between a glycine carbon atom *i* and a water oxygen atom *j* within a cutoff of 10 Å, and *d*_0_ = 2.5 Å.^56^ A bias factor of 15 was used, with Gaussians deposited every 1 ps to gradually drive the ligand unbinding. The Gaussians had widths of 0.15 Å and 0.03 for the distance and solvation collective variables, respectively, and an initial height of 1.2 kJ/mol. The two- and three-ligand models were subjected to a 100 ns MD simulation to equilibrate the structure after ligand ejection, followed by a 1000 ns MD production run for analysis. The models with partial ligand occupancies could have also been prepared by simply removing some ligands from the initial structure; the two procedures are alternative and unlikely to give significantly different results upon reasonably long simulations, as Loop C, which opens when the ligand detaches, becomes flexible, opening and closing easily over the binding site in the absence of the ligand.

The fully unbound Apo structure was also constructed and simulated for the sake of comparison, using the same modeling protocol as that for the bound structure. The Apo model has 216 residues per subunit (two less than the bound structure at the N-terminus), and the solvated system consisted of 125 287 atoms in an initial orthorhombic cell of 114 Å × 114 Å × 114 Å.

We concentrated on the two- and three-ligand systems to compare with the fully ligated and Apo systems, as experiments seem to indicate that two or three ligands are sufficient to activate pLGICs, as previously discussed. The three-ligand system contains both consecutive and nonconsecutive binding pockets, while we first focused on a two-ligand system with nonconsecutive binding pockets. We then built and simulated an additional replica with two ligands, with the two consecutive pockets (d) and (e) occupied. We acknowledge that the selected systems do not exhaust all possible bound patterns; in fact, we did not consider four ligand systems and one ligand system as well as all possible permutations of ligand occupations in the different subunits, which are supposed to be equivalent but subject to fluctuations. However, we believe that the selected systems are representative, if not exhaustive, to study the effects of having bound and unbound pockets in the same ECD of a pLGIC. The five binding sites in the different models represent different scenarios of bound/unbound pockets flanked by combinations of bound/unbound pockets, providing good statistical sampling, although statistics can be improved by running multiple replicas or lengthening the simulation time.

The analysis of the MD trajectories was carried out with the assistance of the Cpptraj^57^ and MDAnalysis software.^58^ Hydrogen bonds were defined by utilizing a 3 Å cutoff for the distance between the donor and the acceptor and a 135° cutoff for the donor–H–acceptor angle. The cation−π interactions were calculated using a threshold of 6 Å for the distance between the cation and the center of the aromatic ring, while the angle between the normal to the ring and the direction joining the center of the ring and the cation was set to less than 45° or greater than 135°.

Root mean square deviations (RMSDs) of the subunits of all models were calculated for the backbone atoms, with respect to the initial frame of the production run. Root mean square fluctuations (RMSFs) were calculated for relevant loops involved in the binding pockets, with respect to the average structure in the production run.

The average number of water molecules inside the pocket was calculated as the average number of the corresponding oxygen atoms enclosed within an “atomic cage” defined by the C_α_ of the 11 binding residues: PHE115, GLU173, SER174, PHE175, TYR218, THR220, PHE223 (in the principal subunit) and PHE79, ARG81, LEU133, SER145 (in the complementary subunit). The SciPy ConvexHull package was used to check if the water oxygens were located inside the cage, which was updated at each MD frame.^59^ The volume of the ConvexHull was also calculated as an estimate of pocket expansion.

To describe the orientation of Loop C, the loop that caps the binding site, we measured the angle defined by the center of mass of the C_α_ of the three pairs of residues describing: the apex of Loop C (residues ASN219 and THR220, indicated with the letter C in Figure 4), its hinge (THR215 and THR224, indicated with O), and a reference point in the complementary subunit (ASN58 and ARG81, indicated with G). This angle has the advantage of considering only the bending of Loop C, disregarding other factors, e.g., Loop B or principal subunit movement; we found it an appropriate descriptor because Loop C had a relatively rigid structure during the simulations, acting as a lid that opens and closes over the binding pocket. In fact, the distance between the apex and hinge of Loop C remained relatively constant during all simulations. Alternative methods employed to assess Loop C include aligning structures to evaluate the degree of capping^17,60^ and monitoring a specific distance between Loop B and Loop C to describe different pocket states.^61^

**Figure 4:**
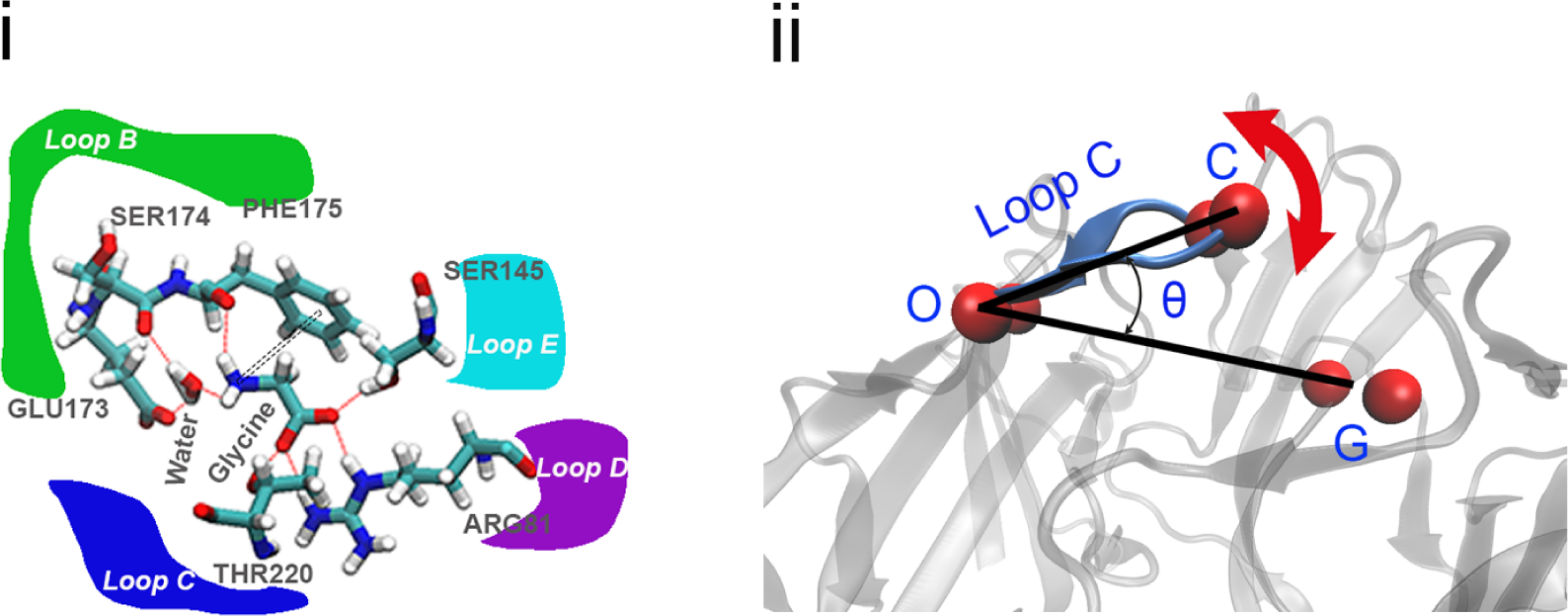
(i) Main interactions between zwitterionic glycine and key binding residues, including hydrogen bonds, water bridges, and cation−π interactions. (ii) Illustration of the angle defining the orientation of Loop C (in blue) with respect to the protein structure.

Protein contact maps were generated to identify the relevant interactions between adjacent subunits. The 2 × 2 contact maps represent the occurrence of distances below 11 Å between the C_α_ of pairs of amino acid residues of adjacent principal and complementary subunits, averaged over MD production runs.

## Results and Discussion

All MD simulations were stable with backbone RMSD per subunit of about 2 Å as reported in Figure S1. All glycines remained bound, although the ligand in pocket (a) of the all-ligand model became loosely bound toward the end of the simulation and would unbind had the simulation been continued. This was confirmed by subsequent enhanced sampling simulations, which used the final structure of the all-ligand model MD as their initial structures.^62^

Upon binding, the binding pocket contracts due to Loops C and B moving toward the agonist; the pocket volume is reduced by squeezing out water molecules.^17^ Previous experimental work on AChRs pointed out that each binding site can undergo low-affinity (LA) ↔ high-affinity (HA) switching, indicative of dynamical behavior and linked to Loop C capping.^61^ In our models, the occupied pockets have volumes smaller than those of the empty pockets. However, there are also some variations in the volume of bound (and also unbound) pockets for different binding sites within the same and the other models, as also reflected by other correlated quantities, such as the presence of water molecules and the orientation of Loop C. This will be quantified and discussed in detail later.

The binding site of GlyR includes a diverse range of residues, which consistently interact with glycine ligands mainly through hydrogen bonds (including water-bridged ones) and cation−π interactions. The occurrence (accounting whether a residue interacts with glycine), average number (including also the possibility of multiple bonds with the same residue), and corresponding standard deviation of hydrogen bonds, water-bridged hydrogen bonds, and cation−π interactions are reported in Tables 1–3, respectively. A typical bonding pattern with key residues is shown in Figure 4.

**Table 1:** Occurrence (In %) of Hydrogen Bonds between the GlyR Residues and Glycine in the Bound Pockets of the Models; in the Last Column, the Total Average Number of Hydrogen Bonds and Its Standard Deviation Are Reported^a^

Model	Pocket	ARG81	SER145	THR220	PHE175	Total Number
Full	a	92.2	91.8	77.2	66.2	4.0 ± 1.0
	b	92.7	93.2	81.6	48.4	3.9 ± 0.9
	c	92.8	92.1	80.8	51.4	3.9 ± 0.9
	d	94.2	96.2	81.6	61.5	4.0 ± 0.9
	e	93.0	93.9	78.9	45.8	3.8 ± 0.9
Three	b	89.2	96.0	79.3	31.9	4.1 ± 0.9
	c	82.7	87.3	63.1	11.0	3.4 ± 0.9
	e	91.9	90.0	24.3	35.7	3.6 ± 0.9
Two	c	93.6	93.7	82.0	36.4	3.8 ± 0.9
	e	93.9	95.3	89.2	52.8	4.1 ± 0.8

a Interactions with occurrence smaller than 2% are not shown.

**Table 2:** Occurrence (In %) of Water-Bridged Interactions between the GlyR Residues and Glycine in the Bound Pockets of the Models^a^

Model	Pocket	GLU173	SER174	PHE115	PHE79	ARG81	THR220	PHE175	TYR218	Total Number
Full	a	75.3	70.3	57.3	6.2	5.8	5.4	1.7	1.4	2.8 ± 0.8
	b	90.2	86.9	4.2	0	0	5.0	0	7.7	2.0 ± 0.5
	c	76.0	71.5	0	0	0	4.7	1.9	27.5	2.3 ± 0.7
	d	78.4	74.6	0	1.3	1.2	5.0	1.2	78.5	2.9 ± 0.5
	e	63.7	67.5	0	7.3	6.3	5.7	0	10.1	2.1 ± 0.7
Three	b	72.7	68.2	4.9	0	0	0	0	2.7	2.0 ± 0.4
	c	59.4	53.8	0	0	0	5.0	26.6	18.4	2.6 ± 1.0
	e	10.8	16.0	0	18.1	29.6	23.3	1.6	16.6	2.0 ± 0.9
Two	c	64.4	60.8	0	0	0	1.1	0	15.8	2.2 ± 0.5
	e	58.7	57.8	0	5.3	0	0	0	7.1	2.1 ± 0.5

a In the last column, the total average number of water-bridged interactions and its standard deviation are reported.

**Table 3:** Occurrence (In %) of Cation−π Interactions between the GlyR Residues and Glycine in the Bound Pockets of the Models^a^

Model	Pocket	PHE79	PHE175	TYR218	PHE223	Total Number
Full	a	10.5	98.3	16.4	30.6	1.6 ± 0.7
	b	2.0	99.9	58.7	52.1	2.1 ± 0.7
	c	5.1	97.6	29.8	35.5	1.7 ± 0.7
	d	4.5	99.9	34.1	33.1	1.7 ± 0.7
	e	1.3	99.9	76.1	41.5	2.2 ± 0.7
Three	b	2.8	99.7	26.2	38.5	1.7 ± 0.7
	c	15.7	97.1	57.9	59.6	2.3 ± 0.9
	e	2.3	98.9	86.7	29.7	2.2 ± 0.6
Two	c	4.1	98.3	18.2	32.1	1.5 ± 0.7
	e	4.1	99.9	50.2	46.3	2.0 ± 0.8

a In the last column, the total average number of cation−π interactions and its standard deviation are reported. Interactions with occurrence smaller than 2% are not shown.

The zwitterionic glycine acts as a bridge across adjacent subunits by interacting with the side chains of two residues with opposite charge: GLU173 (Loop B) and ARG81 (Loop D)^63^ through its charged groups. In our MD simulations, the glycine carboxylate group displays persistent interactions with the positively charged ARG81 forming a salt-bridge (often with two bonds, as shown in Figure 4i). Interestingly, the glycine ammonium group interacts with the negatively charged GLU173 carboxylate not directly, but through a water bridge, which helps stabilize the binding. Other persistent direct hydrogen bonds are formed with SER145 (Loop E), THR220 (at the apex of Loop C, effectively locking this loop over the binding pocket), and PHE175 (Loop B), which also plays an important role in cation−π interactions. SER174 (Loop B) predominantly participates in a water-bridged interaction with glycine, involving the same water molecule that mediates the interaction with GLU173, as shown in Figure 4i. This is consistent with previous MD^64,65^ and structural imaging work.^66^ However, an increase in the amount of water in the binding pocket may disrupt, rather than enhance, the interactions between the ligand and the binding residues; in some of the binding pockets, several other water-bridged interactions occur, often short-lived. Hence, the number of water molecules in the binding pockets is a useful indicator of binding stability.

The average numbers of water molecules in the (bound and unbound) pockets are shown in Figure 5i. In the full-ligand model, pocket (a) includes a slightly higher average number of water molecules, with a value of 1.4 and a significant standard deviation. This is due to the fact that glycine in pocket (a) became loosely bound toward the end of the MD, with a significant increase in the water number (as evident in the time-evolution of the water number in Figure S2). The remaining pockets consistently maintain an average water number of 1.1 with sporadic fluctuations of over 1. In the three-ligand model, the average number of water molecules inside the bound pockets varies from 1.0 in (b) to 1.6 in (c) and 2.1 in (e), while the empty pockets contain 6.4 (a) and 7.6 (d) waters, respectively. In the two-ligand model, pockets (c) and (e) contain on average 1.0 water molecule with a small standard deviation, with a single water molecule inside the pocket for most of the time, while the empty pockets contain 7.5 (a), 8.7 (b), and 7.6 (d) waters, respectively. In the two-ligand model, the nonconsecutive bound pockets (c) and (e) are adjacent to unoccupied pockets on both sides, while in the three-ligand model, only one bound pocket is flanked by unoccupied binding pockets on both sides and none in the all-ligand model. The quantity of water molecules in the bound pockets may be affected by the number of empty pockets and the different competing interactions at the bound and unbound interfaces between subunits. Interestingly, in the Apo structures, the average number of water molecules is higher in the three pockets (a), (c), and (e), at 12.1, 14.6, and 10.1, respectively, and significantly lower in (b) and (d), at 5.0 and 6.0, respectively, showing a tendency to have two nonadjacent pockets more contracted, even in the absence of ligands. This is reflected also in the “convex hull” volume used to identify the water within the binding pockets in Figure 5iii. Interestingly, this self-organization resembles the case of the two-ligand model, if we consider the space occupied by glycine (roughly equivalent to five water molecules) as observed in Figure 5i, suggesting a natural tendency to a favorable assembly where two of the five binding pockets are more contracted than the others in a nonconsecutive pattern, helped or not by the presence of glycines.

**Figure 5:**
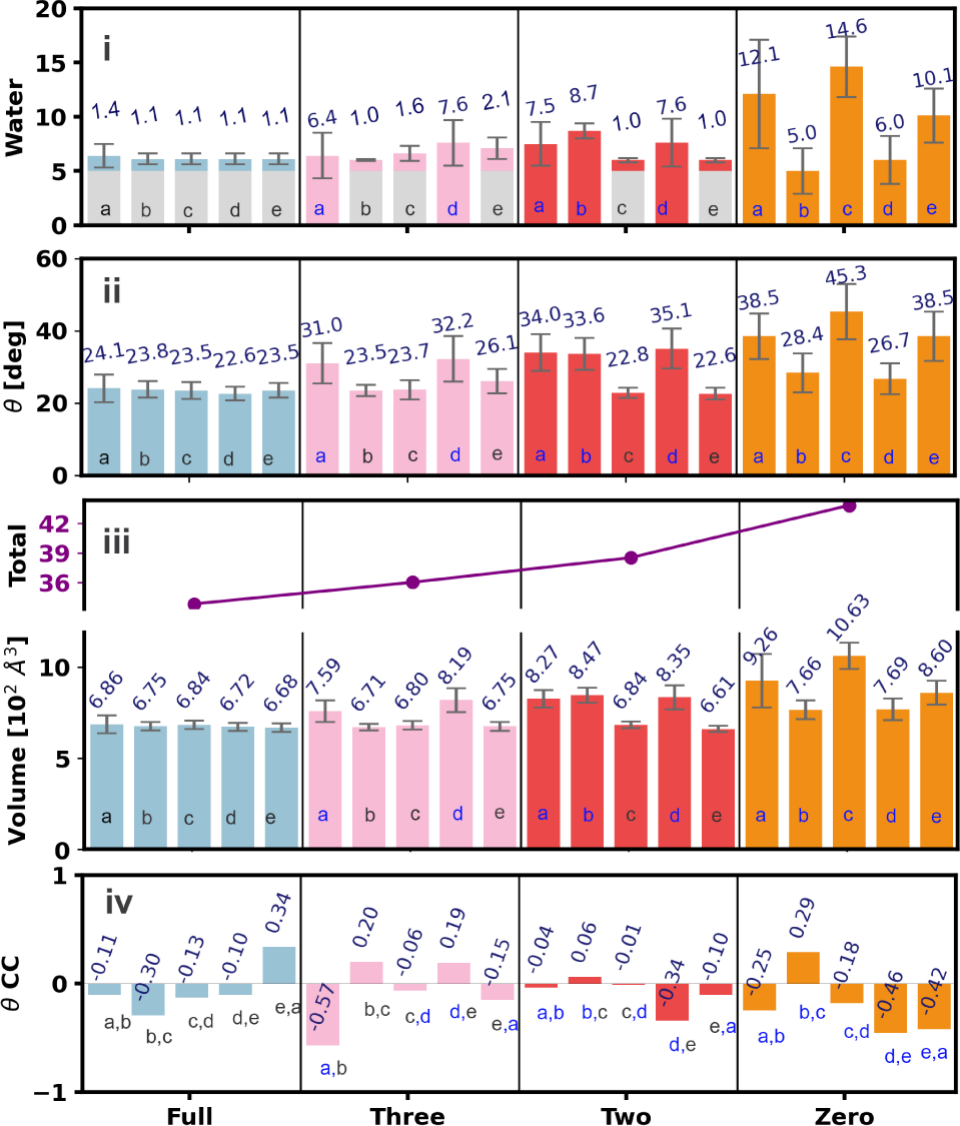
(i) The average number of water molecules in both occupied and unoccupied binding pockets. Values for occupied pockets are raised by 5 to somehow account for the presence of glycine and make them visually comparable to the unoccupied pocket contents. (ii) The average angle (in degrees) representing the orientation of Loop C across all pockets. (iii) The volume of the convex hull, serving as an approximate measure of pocket volume, with the purple line representing the sum of the five pocket volumes for each model. (iv) The correlation coefficient between the orientation angles of Loop C of adjacent pockets.

The water-bridge analysis in Table 2 shows that, when more than one water molecule is present in the binding pockets, glycine is able to form a water bridge with several residues (e.g., in the full-ligand model pocket (a), three-ligand model pocket (c), and (e)) with reduced occurrence and less stable interactions, likely weakening the overall binding stability with respect to when a single water molecule, bridging the interaction with GLU173 and SER174, is present. Therefore, there is a subtle balance between the stability of glycine binding and the water content in the binding pockets.

The binding pocket is lined by several aromatic residues, many of which are conserved among the pLGICs. These residues typically form an “aromatic cage” that helps the pocket recognize ligands and stabilize ligand binding through cation−π interactions, in addition to hydrogen bonds.^67–69^ Upon the binding of glycine, in all models, about two cation−π interactions are formed. In this context, the most important aromatic residue is PHE175 (in Loop B), which forms cation−π interactions with high occurrence (over 97%) and participates as a donor in hydrogen bonding (with varying occurrences ranging from 11% to 66%). TYR218 and PHE223 (in Loop C) and PHE79 (in Loop D) can also participate in cation−π interactions, although less frequently (with varying occurrences in the ranges of 16–87%, 30–60%, and 1–16%, respectively).

The bound pockets with larger water content, namely, pockets (c) and (e) in the three-ligand model, as well as pocket (a) in the full-ligand model, exhibit decreased interactions between the ligand and THR220 at the apex of Loop C. In fact, the average number of water molecules in the pockets strongly correlates with the orientation of Loop C, which caps the binding site upon glycine binding and opens upon unbinding or in the absence of glycine. Upon entering the pocket, glycine interacts with THR220 in Loop C and drags this loop to cover the binding pocket, as also shown in other Cys-loop receptor simulations,^45^ resulting in a contraction of the pocket.^17,61^

There are a wealth of mutagenesis experiments that detail a variety of changes mediated by both artificial and natural alterations in the genes for GlyRs in the broader context of hyperekplexia. Some of the investigated mutations directly affect glycine binding in the ECD. From the residues discussed in this work (translating to the corresponding residue numbering, which is consistent with that of the paper with structures^17^), ARG81 has been identified many times as critical for ligand binding: the mutation of this residue often led to complete cessation of current generation in response to glycine application in the cases where trafficking and expression were not also impeded. A residue close to the binding pocket, GLU119, was found to drastically increase EC50 but did not have an effect on maximum current generation, tying the role of this residue closely to the binding process itself.^70^ In similar work probing the effects of mutants in a heteromeric GlyR in the α subunits, Glu173 was identified as a key residue mediating intersubunit interactions, evidenced by data that demonstrate substantial drops in apparent binding affinity. ARG147 was mentioned but mainly in relation to its impact on the binding of the antagonist strychnine. ARG135 was found to have similar drastic effects on the affinity of the ligand to ARG81, with the reasoning attributed to proximity to the binding pocket and impacts on binding alone, especially when considering the lack of impact on gating or maximal currents.^63^

The orientation of Loop C was monitored through the angle shown in Figure 4ii and described in the Methods section; results for all models are shown in Figure 5ii; the time-evolution can be found in Figure S3. In the ligated systems, angles in the range 22–26° are associated with bound pockets, while those associated with unbound pockets are in the range 31–35°. The strong correlation between the water content in the binding pocket and the orientation of Loop C is evident in Figure 5ii: e.g., pocket (a) of the full-ligand model has the largest Loop C angle value as well as the largest average water content; similarly, pocket (e) of the three-ligand model has the largest angle and the largest water content among the bound pockets.

In the Apo state, all pockets are empty, enabling the ligand, if present, to enter the pockets, although, interestingly, with an alternate pattern of angles: three in the range 38–45° (a, c, e) and two 27–28° (b, d), corresponding to the much lower water content in these pockets and the contracted volumes previously discussed. These more “closed” (more compressed) pockets behave in a manner not dissimilar to that of the more “open” (more enlarged) bound pocket in the other models (with an equivalent content of water or water plus ligand). This may indicate a mechanism of conformational selection,^71^ where the level of contraction/expansion of the pockets may facilitate or hinder ligand binding, contributing to favorable binding patterns in GlyRs and other pLGICs sharing structural similarities.

The homomeric GlyR has five subunits assembled symmetrically around the axis of the ion channel. The ligands at the interfaces between the subunits may induce competing push–pull interactions. The fact that the subunits are five, an odd number, may produce some degree of frustration, possibly making the two-ligand systems with nonconsecutive occupation more suitable for binding site contraction and favorable binding. The alternate pattern of the orientation angle of Loop C and the water content in the pockets of the Apo model as well as in the ligated systems suggest that there may be synergistic interactions among subunits. However, the correlation between the state of “openness” of Loop C of different pockets is difficult to capture. For example, the Loop C orientation angle of each pocket was correlated to that of the adjacent pocket and quantified with the Pearson correlation coefficient, as shown in Figure 5iv. The values are small and do not appear to show clear patterns; however, they only assess fluctuations, whether the Loop C angles of adjacent subunits tend to increase or decrease simultaneously, rather than the propensity of the pockets to be more or less open.

To assess the behavior of GlyR residues in the loops of the binding pockets as a function of ligand occupancy, root mean square fluctuations were calculated for each principal subunit in all models and reported in Figure 6. It was previously suggested that the highest levels of fluorescence at residue ALA68 of Loop 2 were directly related to the ability of an agonist to bind and function.^72^ We found that when glycine is bound to the pocket, the fluctuations of residues in Loop 2 increase with the largest RMSF observed for THR70, not far from ALA68. Residues in Loop C show increased RMSF for the empty pockets, as expected. Overall, these are the largest differences observed in the RMSF analysis of the ligated systems. In the Apo model, pockets (a), (c), and (e) show a larger RMSF for Loop C in comparison to pockets (b) and (d). This is consistent with the previous analysis of the Loop C orientation angle and water content. An interesting behavior is shown by Loop B; the RMSFs of Loop B residues tend to have a peak at GLY176 in the empty pockets in both the Apo and the ligated models: this residue is adjacent to the triplet GLU173, SER174, and PHE175, which are involved in interactions with the ligand when bound, thus hindering its fluctuations. All loops, except Loop 2, tend to have larger RMSF values in the Apo model.

**Figure 6:**
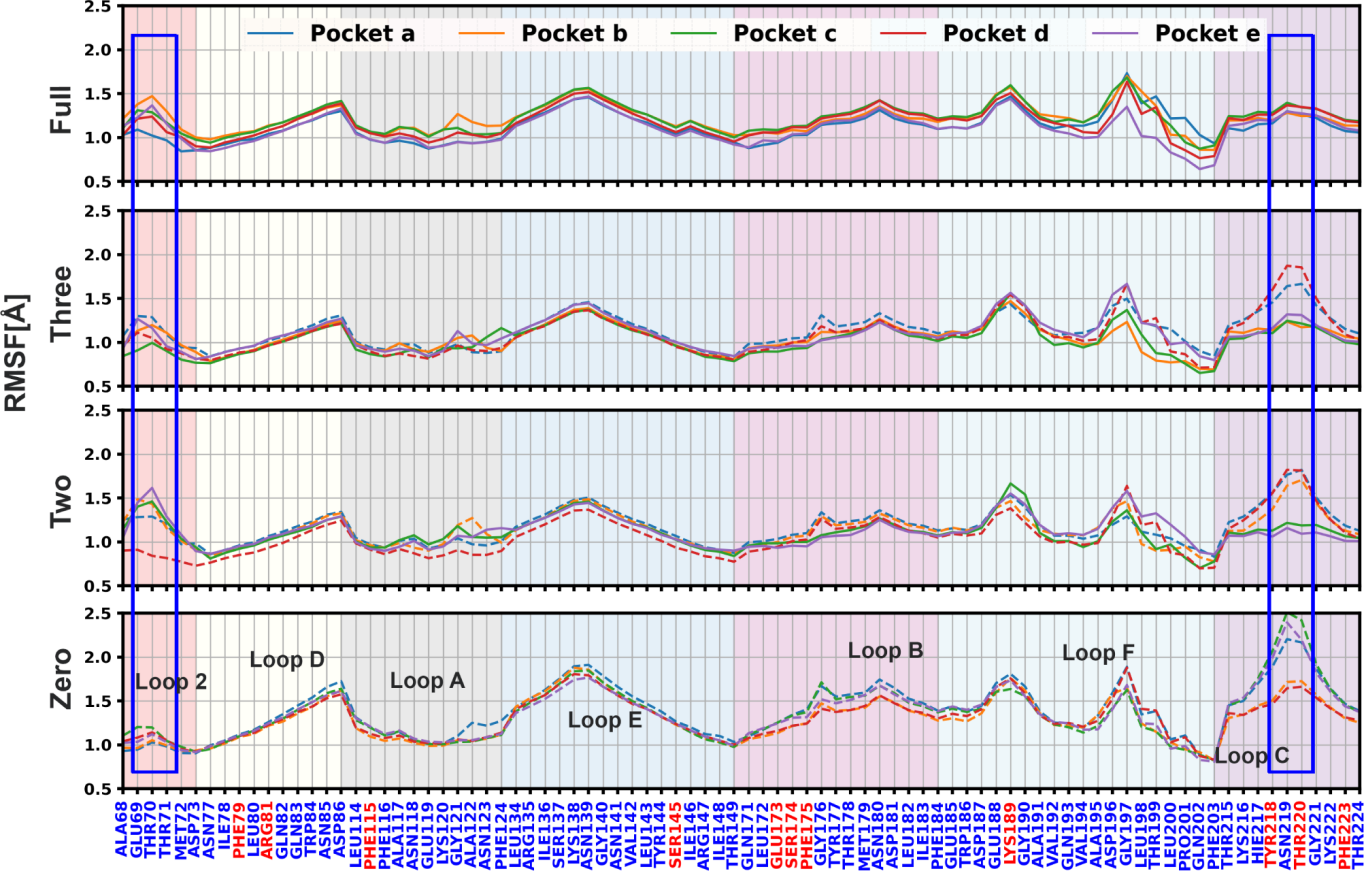
Root mean square fluctuations (RMSFs) of residues of the loops forming the binding pocket of all subunits of all models, including Loops A, B, C, D, E, F, and 2 from the principal subunit of a pocket. The colored backgrounds indicate the different loops; solid lines are for bound pockets, and dashed lines are for empty pockets. Binding residues are highlighted in red.

The indicators of pocket contraction (e.g., water content, Loop C angle, and convex hull volume) may correlate with subunit movements. These were analyzed by examining the distributions of the average positions of the C_α_s of the binding residues within each subunit, as shown in Figure 7 and Table 4. The zero-ligand model displays larger fluctuations in the distributions in comparison with the other models, indicating greater subunit movement and fluctuations in the Apo state pockets. The distances between the averages of the distributions are shown in Figure 7. In the bound models, the intrapocket distances exhibit patterns similar to the water content, Loop C angle, and convex hull volume analysis: bound pockets tend to display shorter intrapocket distances (on average 11.6 ± 0.2 Å) than unbound pockets (on average 12.4 ± 0.3 Å), showing the pulling effect of the ligand with respect to the principal and complementary subunits. The presence or absence of this pulling effect on either side of each subunit also affects the interpocket distances. In fact, these distances are on average the largest (16.7 ± 0.1 Å) between bound pockets, followed by those between a bound and an unbound pocket (16.5 ± 0.1 Å), with the shortest distance of 15.8 Å observed between unbound pockets. While these differences are very subtle and affected by uncertainties, they relate to the C_α_ positions, which are fairly stable and are indicative of a push–pull mechanism for subunits depending on the presence or absence of a ligand at the interfaces to pull them toward the adjacent subunit. The Apo structure shows a variety of distances reflecting the alternation of more or less expanded unbound pockets and protein self-organization, with an average intrapocket distance of 13.0 ± 1.3 Å and an average interpocket distance of 16.4 ± 0.2 Å. In this case, there are no ligands to connect the subunits, which have slightly different tilted structures with respect to the bound structures. Hence, it is difficult to make a direct comparison, but what is interesting is to observe, in the absence of ligands, the different contractions and expansions of the binding pockets, which may facilitate or hinder selected binding.

**Figure 7:**
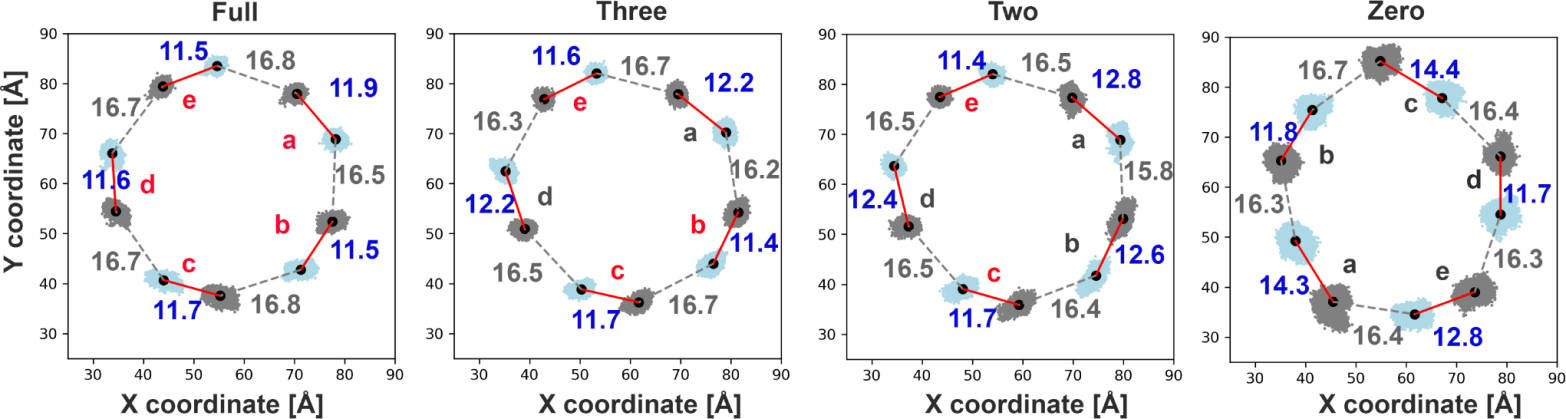
Positions of the centers of mass of the binding pockets in the *X*–*Y* plane orthogonal to the channel axis are shown. The pockets are labeled (a–e) for each model. For each pocket, the two scatter distributions represent the centers of mass of the C_α_ of the binding residues, respectively, on the principal (light blue) and on the complementary (gray) subunits that form the pocket. The labels of unbound pockets are in gray, while those of bound pockets are in red. Gray dashed lines connect scatter distributions in the same subunit, indicating interpocket distances (values in Å shown in gray), while red solid lines represent intrapocket distances (values in Å shown in blue).

**Table 4:** Intrapocket (a, b, c, d, and e) and Interpocket (a-b, b-c, c-d, d-e, and e-a) Distances (In Å) in the Investigated Models^a^

Model	a	a-b	b	b-c	c	c-d	d	d-e	e	e-a
Full	**11.9**	⟨16.5⟩	**11.5**	⟨16.8⟩	**11.7**	⟨16.7⟩	**11.6**	⟨16.7⟩	**11.5**	⟨16.8⟩
Three	12.2	16.2⟩	**11.4**	⟨16.7⟩	**11.7**	⟨16.5⟩	12.2	16.3⟩	**11.6**	⟨16.7
Two	12.8	15.8	12.6	⟨16.4⟩	**11.7**	⟨16.5	12.4	16.5⟩	**11.4**	⟨16.5
Zero	14.3	16.3	11.8	16.7	14.4	16.4	11.7	16.3	12.8	16.4

a Intrapocket distances of bound pockets are in boldface. ⟨ and ⟩ indicate participation to the bound pocket “on the left” and/or “on the right”.

The movement of subunits is influenced by both ligand action in attracting adjacent subunits and protein self-organization, which can be reflected in intersubunit interactions. In the ligated systems, the number of direct intersubunit hydrogen bonds obtained by summing the average number for the five subunits (Figure S4) slightly increases as the number of bound ligands decreases from five (42.6 ± 1.8) to three (43.7 ± 2.0) to two (44.7 ± 2.1). However, the more ligands that are bound, the more additional interactions mediated by the ligands that are present, as previously discussed, strongly enhancing the adhesion of adjacent subunits. The Apo structure shows a substantially reduced number of intersubunit hydrogen bonds (32.4 ± 2.4) with respect to the ligated models, consistently with a less compact structure, with subunits in the ECD undergoing an outward tilt from the channel axis when transitioning from the open/desensitized state to the Apo state.^73^

Some interactions involving specific residues observed in the ligated models are still detectable in the Apo model, although with lower occurrences. In the zero-ligand model, some interface residues often form interactions with multiple residues on the adjacent subunit instead of a single residue with high occurrence. Selected hydrogen bonds between conserved residue pairs across bound pockets in the ligated models are shown in Figure S5. These interactions are no longer maintained in the zero-ligand model except for the salt bridge between GLU119 (Loop A) and ARG147 (Loop E) and, to a lesser extent, for that between LYS49 and ASP96. The GLU119-ARG147 salt bridge appears essential for stabilizing the ECD structure and has been suggested to be a key determinant of agonist efficacy, with its influence being agonist-dependent;^66^ its location in the protein is shown in Figure S6. Additional notable interactions include those between Loop C and residues on the complementary subunit (residue pairs: ASN219-ASN58 and THR220-ARG135), commonly observed in bound pockets due to the attraction between glycine and Loop C.

The intersubunit interactions were further analyzed using protein distance contact maps, as shown in Figures S7 and S8. Two distinct regions, labeled “Region 1” and “Region 2” in Figure S7, display notable differences between bound and unbound pockets and across ligated models versus the Apo model. “Region 1” includes interactions between Loop C of the principal subunit and residues on the complementary subunit with weakened interactions in unbound pockets. Focusing specifically on the interactions between Loop C on the principal subunit and Loop F on the complementary subunit, Figure S8 reveals that unbound or “more enlarged” pockets typically exhibit weakened interactions between (+, Loop C)­ASN219 and (−, Loop F)­LYS189, while bound or “more compressed” pockets tend to show enhanced interactions at this site. In the zero-ligand model, potential interactions in “Region 2” are absent due to the tilting of the subunit in the Apo state.

Loop F participates in a pocket as part of a complementary subunit but is covalently linked to Loop B in the same subunit. Loop B, in turn, participates in the adjacent binding site as part of the principal subunit and interacts with Loop E of the following complementary subunit, as shown in Figure 8.

**Figure 8:**
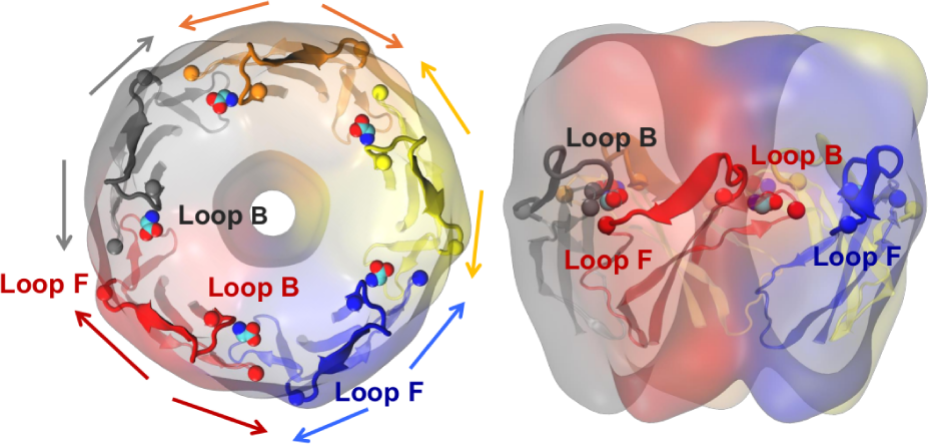
Top (left) and side (right) views of the GlyR ECD with subunits shaded in different colors. The connected Loop F and Loop B are shown for each subunit and explicitly labeled for two adjacent occupied binding sites. The suggested push or pull effects of the occupied binding sites are indicated by arrows.

The movements of these structures are therefore connected and can be quantified by monitoring the distances involving the representative residues. In Figure 9, we consider three consecutive subunits: for clarity, in the following, the first is unlabeled (shown in gray), the second is labeled with ′ (in red), and the third with ″ (in blue). The selected residues (within the respective loops) are (+, Loop C)­ASN219, (−, Loop F′)­LYS189, (+, Loop B′)­SER174, and (−, Loop E″)­SER145. As previously mentioned, the ASN219-LYS189 distance, involving Loop C and Loop F′ of the first two subunits, is generally shorter in bound or “more compressed” pockets, reflecting a stronger interaction. Likewise, the distance between (+, Loop B′)­SER174 and (−, Loop E″)­SER145 is shorter for bound or “more compressed” pockets, compared to unbound or “more enlarged” pockets, as shown in Figure 9ii. This distance distribution suggests that the movement of Loop B′, determined by the presence or absence of a ligand in the pocket, may influence that of LYS189 on Loop F′, to which it is covalently connected. To monitor this, the distributions of the distance between (+, Loop F′)­LYS189 and (−, Loop E″)­SER145 are also shown in Figure 9. The two distances always show a positive correlation, which is stronger in unbound (less compressed) pockets (Figure 9iii), indicating that the movement of Loop B′ transmits effectively to Loop F′, especially when there are no restrictions, but can be affected by the pulling action of glycine mediating the interaction between Loop B′ and Loop E″. In turn, LYS189, located on the Loop F′ protrusion, forms part of the complementary side of the pocket between the first two subunits and is positioned near Loop C of the principal side of that pocket, as shown in Figure 9i. When the corresponding pocket is occupied, Loop C, where ASN219 is located, acts as a lid to cover the pocket, while the protrusion of Loop F′, including LYS189, may act as a secondary lid that reinforces Loop C capping. The state of adjacent pockets appears to influence this secondary lid, suggesting a cooperative interaction between pockets through a “push–pull” mechanism.

**Figure 9:**
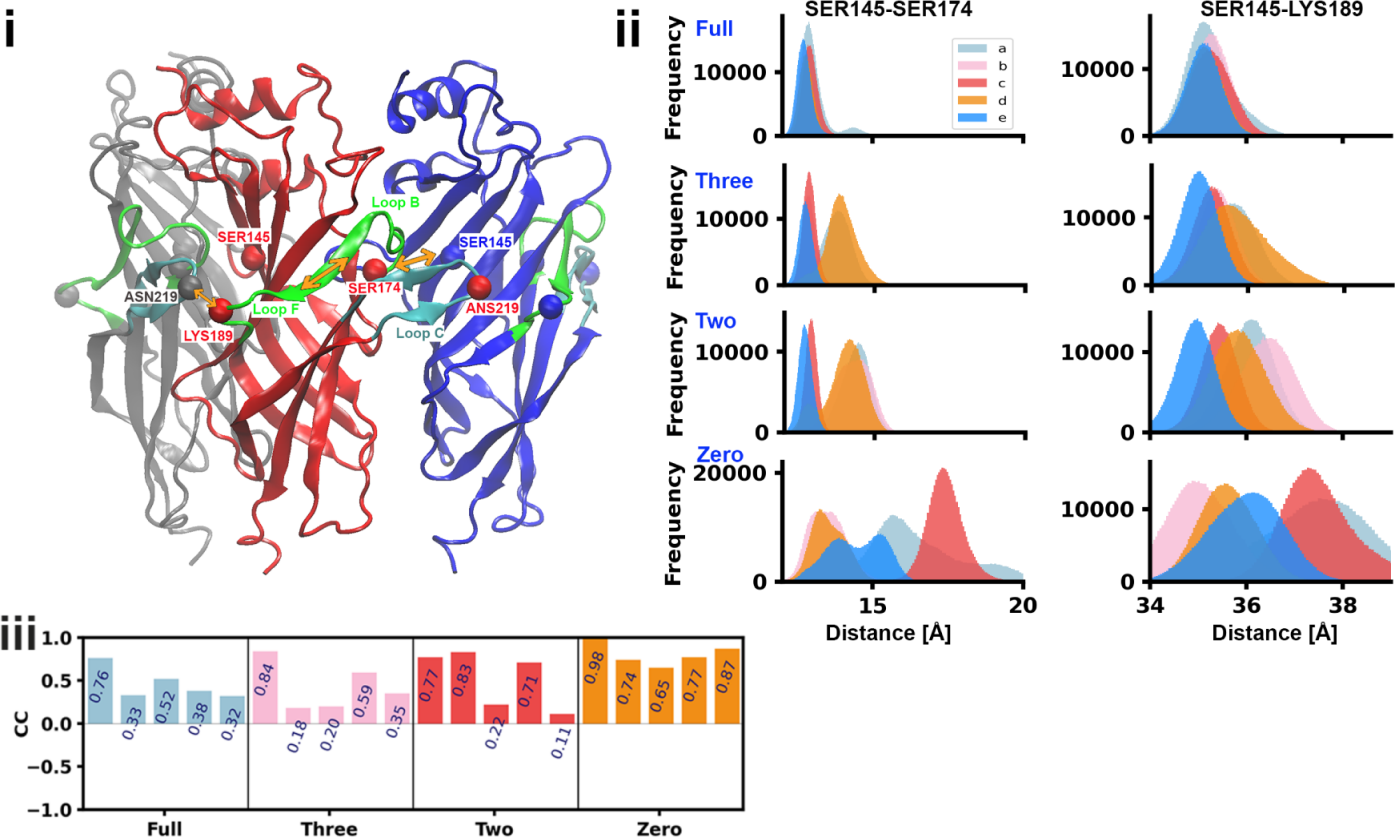
(i) Representation of three consecutive subunits, with the C_α_s of ASN219, LYS189, SER174, and SER145 represented as colored spheres. Loop B′ and F′ are in green. (ii) Histogram of the distribution of the distances between residues SER174 (+, Loop B′) and SER145 (−, Loop E″) and between LYS189 (+, Loop F′) and SER145 (−, Loop E″). (iii) Correlation coefficient between the distances SER174-SER145 and LYS189-SER145 across pockets in each model. In the three-ligand model, the bound pockets are (b), (c), and (e); in the two-ligand model, the bound pockets are (c) and (e).

The results for an alternative two-replica model are reported in Figures S9–S13 and Table S1. Similar features as for the model discussed here are observed, related to the water content and orientation of Loop C. In the case of the occupation of two consecutive pockets, there seems to be a tendency to have “more closed” pockets, in particular, the unbound pocket flanked by unbound pockets, reminiscent of the alternation seen in the Apo structure. If this is a signature of conformational selection, it may imply the favorable binding of a third ligand.

## Conclusions

Homomeric pLGICs are symmetrically assembled with five identical subunits, showing a synergistic cooperation that facilitates channel gating. Despite extensive computational and experimental research, the nature of this intersubunit cooperation remains incompletely understood. If subunits cooperate, the bound or unbound status of each pocket would influence the status of adjacent pockets. To explore this, we focused on a prototypical pLGIC, the homomeric α1 GlyR, which is activated by the binding of zwitterionic glycine. Inspired by the knowledge that two or more bound glycine molecules are sufficient to efficiently activate the receptor, we constructed four GlyR-glycine ECD models with varying numbers of bound glycine ligands (full-, three-, two-, and zero-ligand (Apo) models) and performed molecular dynamics simulations on them. This simulation design enabled analysis across four distinct and representative scenarios: bound pockets adjacent to bound pockets, bound pockets adjacent to one or two unbound pockets, and unbound pockets adjacent to other unbound pockets.

The binding interactions between glycine and key residues in each bound pocket across all models were thoroughly analyzed, showing how glycine, when present, connects adjacent subunits, contracts the binding pocket, and ejects excess water molecules. Glycine key interactions include hydrogen bonds with ARG81 (−, Loop D), SER145 (−, Loop E), THR220 (+, Loop C), and PHE175 (+, Loop B); water bridges with GLU173 (+, Loop B) and SER174 (+, Loop B); and cation−π interactions with PHE175 (+, Loop B). Notably, ARG81 plays a critical role, forming a salt bridge with the carboxylate group of glycine, and GLU173 interacts with the ammonium group of glycine through a water bridge, while PHE175 can simultaneously form hydrogen bonds and cation−π interactions with glycine.

Interestingly, an optimal number of water molecules must be present to ensure efficient binding, helping glycine to connect the principal and complementary subunits. In fact, an increase in the water content of the binding pocket can disrupt or even replace direct interactions between glycine and the binding residues, resulting in weaker binding; water molecules tend to fill up unoccupied pockets. The quantity of water in each pocket correlates strongly with the orientation of Loop C, which acts as a lid covering the binding pocket when the ligand is inside as well as with pocket volume. A larger number of water molecules in the pocket is associated with a larger Loop C orientation angle, a signature of a more open lid, allowing the ligand to translocate in or out, and with a larger pocket volume.

Across all models, pockets exhibit different levels of water content, Loop C orientation, pocket volume, and RMSFs. The variance among pockets is mainly due to the action of glycine binding: bound and “more compressed” pockets often show enhanced stability, while unbound and “more enlarged” pockets tend to behave in a more unstable manner, with increased fluctuations. The presence of the agonist mediates and enhances intersubunit interactions, attracting the principal and complementary subunits and reducing the space in between. However, we observed that an alternation of “compressed” and “expanded” pockets also developed when no ligand is present in the Apo structure; this is due to self-organization and structure optimization of the five subunits, compatible with frustration effects due to the odd number of subunits, which may be indicative of conformational selection mechanisms.

Either way (regulation by ligands or self-organization), subunit assembly and intersubunit interactions (mediated or not by ligands) play an important role. Subunit assembly was characterized by monitoring the distances between the groups of residues involved in the binding pockets in the principal and complementary subunits in the plane orthogonal to the channel direction. Although within the limitations of the calculations and their uncertainties, we observed a combination of pulling and pushing effects, which are induced by the action of the ligands (and to some extent self-organization) “zipping” together adjacent subunits. We further examined intersubunit interactions, through hydrogen bond analysis and contact maps. In particular, we focused on how covalently linked structures, like Loop F and Loop B, within the same subunit bridge two adjacent binding sites and mediate the suggested push–pull action.

The comparison between the two-ligand models (with consecutive and nonconsecutive pocket occupancies) revealed that the two bound pockets remained stable regardless of their arrangement. Interestingly, the three consecutive empty pockets exhibited an alternation of enlargement (for the side pockets) and contraction (for the central ones), which is likely due to a self-organization tendency of the subunits and potentially conformational selection mechanisms.

The simulations presented in this work provide detailed molecular-level insights into the dynamics of bound and unbound binding pockets in the ECD of the glycine receptor and their potential interplay, complementing structural and functional experimental information. The “push–pull” mechanisms suggested here are likely to be relevant to other pLGICs, which are characterized by similar structural features with ligand binding across subunit interfaces. Ligand-specific characteristics (e.g., in GlyR, the way glycine makes use of water to optimize its binding) may also play a tuning role. Heteromeric channels may exhibit specific behaviors at the different interfaces, with the potential to enhance asymmetry.^11–14^

Limitations of our study include the choice of the structures for building the simulation models, the selection of representative but not exhaustive binding patterns, the timescale, and the statistics. Moreover, we exclusively focused on the ECD, disregarding the coupling with the TMD and related activation free energy barriers, which were previously examined at a coarse-grained level.^28^ This coupling, which translates ligand-binding effects to channel gating, is ultimately crucial to obtaining a complete picture of pLGIC function, but it is still very challenging to simulate in realistic detail.

## Supplementary Material



## Data Availability

The data supporting this article were generated using NAMD 2.14 ^50,51^ and PLUMED.^54,55^ They were analyzed with Cpptraj^57^ and MDAnalysis.^58^ They are openly available in the King’s Open Research Data System (KORDS) and can be accessed at https://doi.org/10.18742/28510367.

## Notes

RMSD analysis, time-evolution of water content in the binding pockets, Loop C orientation, and intersubunit hydrogen bond numbers; details of the GLU119-ARG147 salt-bridge; contact maps; analysis of the additional two-ligand replica (PDF)

## References

[1] Hernandes M. S., Troncone L. R. (2009). Glycine as a neurotransmitter in the forebrain: a short
review. J. Neural Transm..

[2] Gundersen R., Vaagenes P., Breivik T., Fonnum F., Opstad P. (2005). Glycine–an
important neurotransmitter and cytoprotective agent. Acta Anaesthesiol. Scand..

[3] Bowery N. G., Smart T. G. (2006). GABA and glycine
as neurotransmitters: a brief history. Br. J.
Pharmacol..

[4] Dutertre S., Becker C.-M., Betz H. (2012). Inhibitory
glycine receptors: an
update. J. Biol. Chem..

[5] Avila A., Nguyen L., Rigo J.-M. (2013). Glycine
receptors and brain development. Front. Cell
Neurosci..

[6] Breitinger U., Breitinger H.-G. (2020). Modulators of the inhibitory glycine
receptor. ACS Chem. Neurosci..

[7] Corringer P.-J., Poitevin F., Prevost M. S., Sauguet L., Delarue M., Changeux J.-P. (2012). Structure and pharmacology
of pentameric receptor channels:
from bacteria to brain. Structure.

[8] Kirsch J., Wolters I., Triller A., Betz H. (1993). Gephyrin antisense
oligonucleotides prevent glycine receptor clustering in spinal neurons. Nature.

[9] Meyer G., Kirsch J., Betz H., Langosch D. (1995). Identification of a
gephyrin binding motif on the glycine receptor *β* subunit. Neuron.

[10] Specht C. G., Izeddin I., Rodriguez P. C., El Beheiry M., Rostaing P., Darzacq X., Dahan M., Triller A. (2013). Quantitative
nanoscopy of inhibitory synapses: counting gephyrin molecules and
receptor binding sites. Neuron.

[11] Liu X., Wang W. (2024). Gating mechanism of
the human *α*1*β* GlyR by
glycine. Structure.

[12] Liu X., Wang W. (2023). Asymmetric gating of
a human hetero-pentameric glycine receptor. Nat. Commun..

[13] Gibbs E., Klemm E., Seiferth D., Kumar A., Ilca S. L., Biggin P. C., Chakrapani S. (2023). Conformational
transitions and allosteric
modulation in a heteromeric glycine receptor. Nat. Commun..

[14] Zhu H., Gouaux E. (2021). Architecture and assembly
mechanism of native glycine
receptors. Nature.

[15] Beato M., Groot-Kormelink P. J., Colquhoun D., Sivilotti L. G. (2004). The activation
mechanism of *α*1 homomeric glycine receptors. J. Neurosci..

[16] Carland J. E., Cooper M. A., Sugiharto S., Jeong H.-J., Lewis T. M., Barry P. H., Peters J. A., Lambert J. J., Moorhouse A. J. (2009). Characterization
of the effects of charged residues in the intracellular loop on ion
permeation in *α*1 glycine receptor channels. J. Biol. Chem..

[17] Yu J., Zhu H., Lape R., Greiner T., Du J., Lu W., Sivilotti L., Gouaux E. (2021). Mechanism of gating and partial agonist
action in the glycine receptor. Cell.

[18] Unwin N. (2013). Nicotinic
acetylcholine receptor and the structural basis of neuromuscular transmission:
insights from Torpedo postsynaptic membranes. Q. Rev. Biophys..

[19] Brejc K., Van Dijk W. J., Klaassen R. V., Schuurmans M., Van Der Oost J., Smit A. B., Sixma T. K. (2001). Crystal structure
of an ACh-binding protein reveals the ligand-binding domain of nicotinic
receptors. Nature.

[20] Galzi J.-L., Revah F., Black D., Goeldner M., Hirth C., Changeux J.-P. (1990). Identification of
a novel amino acid alpha-tyrosine
93 within the cholinergic ligands-binding sites of the acetylcholine
receptor by photoaffinity labeling. Additional evidence for a three-loop
model of the cholinergic ligands-binding sites. J. Biol. Chem..

[21] Dennis M., Giraudat J., Kotzyba-Hibert F., Goeldner M., Hirth C., Chang J. Y., Lazure C., Chretien M., Changeux J. P. (1988). Amino acids
of the Torpedo marmorata acetylcholine receptor. alpha. subunit labeled
by a photoaffinity ligand for the acetylcholine binding site. Biochemistry.

[22] Fu D.-X., Sine S. M. (1994). Competitive antagonists bridge the alpha-gamma subunit
interface of the acetylcholine receptor through quaternary ammonium-aromatic
interactions. J. Biol. Chem..

[23] O’Leary M. E., Filatov G. N., White M. M. (1994). Characterization
of d-tubocurarine
binding site of Torpedo acetylcholine receptor. Am. J. Physiol. Cell Physiol..

[24] Sines S. M., Kreienkamp H.-J., Bren N., Maeda R., Taylor P. (1995). Molecular
dissection of subunit interfaces in the acetylcholine receptor: identification
of determinants of *α*-conotoxin M1 selectivity. Neuron.

[25] Martin M., Czajkowski C., Karlin A. (1996). The contributions of
aspartyl residues
in the acetylcholine receptor *γ* and *δ* subunits to the binding of agonists and competitive
antagonists. J. Biol. Chem..

[26] Prince R. J., Sine S. M. (1996). Molecular dissection
of subunit interfaces in the acetylcholine
receptor: identification of residues that determine agonist selectivity. J. Biol. Chem..

[27] Seino Y., Ohashi N., Kohno T. (2018). The endogenous agonist, *β*-alanine, activates glycine receptors in rat spinal
dorsal neurons. Biochem. Biophys. Res. Commun..

[28] Yan J., Chen L., Warshel A., Bai C. (2024). Exploring the Activation
Process of the Glycine Receptor. J. Am. Chem.
Soc..

[29] Mowrey D., Cheng M. H., Liu L. T., Willenbring D., Lu X., Wymore T., Xu Y., Tang P. (2013). Asymmetric ligand binding
facilitates conformational transitions in pentameric ligand-gated
ion channels. J. Am. Chem. Soc..

[30] Crnjar A., Comitani F., Hester W., Molteni C. (2019). Trans–Cis Proline
Switches in a Pentameric Ligand-Gated Ion Channel: How They Are Affected
by and How They Affect the Biomolecular Environment. J. Phys. Chem. Lett..

[31] Shi S., Lefebvre S. N., Peverini L., Cerdan A. H., Rodríguez P. M., Gielen M., Changeux J.-P., Cecchini M., Corringer P.-J. (2023). Illumination
of a progressive allosteric mechanism mediating the glycine receptor
activation. Nat. Commun..

[32] Crnjar A., Comitani F., Melis C., Molteni C. (2019). Mutagenesis
computer
experiments in pentameric ligand-gated ion channels: the role of simulation
tools with different resolution. Interface Focus.

[33] Comitani F., Melis C., Molteni C. (2015). Elucidating
ligand binding and channel
gating mechanisms in pentameric ligand-gated ion channels by atomistic
simulations. Biochem. Soc. Trans..

[34] Flood E., Boiteux C., Lev B., Vorobyov I., Allen T. W. (2019). Atomistic
Simulations of Membrane Ion Channel Conduction, Gating, and Modulation. Chem. Rev..

[35] Guardiani C., Cecconi F., Chiodo L., Cottone G., Malgaretti P., Maragliano L., Barabash M. L., Camisasca G., Ceccarelli M., Corry B. (2022). Computational methods
and theory for ion channel research. Adv. Phys.:
x.

[36] Zhu Z., Deng Z., Wang Q., Wang Y., Zhang D., Xu R., Guo L., Wen H. (2022). Simulation and Machine Learning Methods
for Ion-Channel Structure Determination, Mechanistic Studies and Drug
Design. Front. Pharmacol..

[37] Şterbuleac D. (2021). Molecular
dynamics: a powerful tool for studying the medicinal chemistry of
ion channel modulators. RSC Med. Chem..

[38] Rao S., Klesse G., Lynch C. I., Tucker S. J., Sansom M. S. P. (2021). Molecular
Simulations of Hydrophobic Gating of Pentameric Ligand Gated Ion Channels:
Insights into Water and Ions. J. Phys. Chem.
B.

[39] Oakes V., Domene C. (2019). Capturing the Molecular
Mechanism of Anesthetic Action
by Simulation Methods. Chem. Rev..

[40] Xu T.-L., Gong N. (2010). Glycine and glycine
receptor signaling in hippocampal neurons: diversity,
function and regulation. Progress Neurobiol..

[41] Lynch J. W., Zhang Y., Talwar S., Estrada-Mondragon A. (2017). Glycine receptor
drug discovery. Adv. Pharmacol..

[42] Melis C., Lummis S. C. R., Molteni C. (2008). Molecular
dynamics simulations of
GABA binding to the GABA*
_C_
* receptor: the
role of Arg104. Biophys. J..

[43] Ashby J. A., McGonigle I. V., Price K. L., Cohen N., Comitani F., Dougherty D. A., Molteni C., Lummis S. C. R. (2012). GABA binding
to an insect GABA receptor: a molecular dynamics and mutagenesis study. Biophys. J..

[44] Comitani F., Cohen N., Ashby J., Botten D., Lummis S. C. R., Molteni C. (2014). Insights into the binding of GABA
to the insect RDL
receptor from atomistic simulations: a comparison of models. J. Comput. Aided Mol. Des..

[45] Comitani F., Limongelli V., Molteni C. (2016). The free energy landscape
of GABA
binding to a pentameric ligand-gated ion channel and its disruption
by mutations. J. Chem. Theory Comput..

[46] Anandakrishnan R., Aguilar B., Onufriev A. V. (2012). H++ (web-based
computational prediction
of protonation states and PK of Ionizable groups in macromolecules). Nucleic Acids Res..

[47] Rajendra S., Vandenberg R., Pierce K., Cunningham A., French P., Barry P., Schofield P. (1995). The unique
extracellular disulfide loop of the glycine receptor is a principal
ligand binding element. EMBO J..

[48] Vogel N., Kluck C. J., Melzer N., Schwarzinger S., Breitinger U., Seeber S., Becker C.-M. (2009). Mapping
of disulfide
bonds within the amino-terminal extracellular domain of the inhibitory
glycine receptor. J. Biol. Chem..

[49] Maier J. A., Martinez C., Kasavajhala K., Wickstrom L., Hauser K. E., Simmerling C. (2015). ff14SB: improving
the accuracy of
protein side chain and backbone parameters from ff99SB. J. Chem. Theory Comput..

[50] Bernardi, R. ; Bhandarkar, M. ; Bhatele, A. ; Bohm, E. ; Brunner, R. ; Buch, R. ; Buelens, F. ; Chen, H. ; Chipot, C. ; Dalke, A. . NAMD User’s Guide, 2020. https://www.ks.uiuc.edu/Research/namd/2.14/ug/.

[51] Phillips J. C., Hardy D. J., Maia J. D. C., Stone J. E., Ribeiro J. V., Bernardi R. C., Buch R., Fiorin G., Hénin J., Jiang W. (2020). Scalable
molecular dynamics on CPU and GPU architectures
with NAMD. J. Chem. Phys..

[52] Wang J., Wolf R. M., Caldwell J. W., Kollman P. A., Case D. A. (2004). Development
and testing of a general amber force field. J. Comput. Chem..

[53] Frisch, M. J. ; Trucks, G. W. ; Schlegel, H. B. ; Gaussian 09 Revision C.01, Gaussian,Inc; Wallingfort CT: 2009

[54] Tribello G. A., Bonomi M., Branduardi D., Camilloni C., Bussi G. (2014). PLUMED 2: New feathers for an old
bird. Comput.
Phys. Commun..

[55] Bonomi M., Bussi G., Camilloni C., Tribello G. A., Banáš P., Barducci A., Bernetti M., Bolhuis P. G., Bottaro S., Branduardi D. (2019). Promoting transparency and reproducibility
in enhanced molecular simulations. Nat. Methods.

[56] Rizzi V., Bonati L., Ansari N., Parrinello M. (2021). The role of
water in host-guest interaction. Nat. Commun..

[57] Roe D. R., Cheatham T. E. (2013). PTRAJ and CPPTRAJ: software for processing
and analysis
of molecular dynamics trajectory data. J. Chem.
Theory Comput..

[58] Michaud-Agrawal N., Denning E. J., Woolf T. B., Beckstein O. (2011). MDAnalysis:
a toolkit for the analysis of molecular dynamics simulations. J. Comput. Chem..

[59] Virtanen P., Gommers R., Oliphant T., Haberland M., Reddy T., Cournapeau D., Burovski E., Peterson P., Weckesser W., Bright J. (2020). Fundamental algorithms
for scientific computing in python and SciPy 1.0 contributors. Nat. Methods.

[60] Maksay G., Laube B., Schemm R., Grudzinska J., Drwal M., Betz H. (2009). Different binding modes
of tropeines
mediating inhibition and potentiation of *α*1
glycine receptors. J. Neurochem..

[61] Jadey S., Auerbach A. (2012). An integrated catch-and-hold
mechanism activates nicotinic
acetylcholine receptors. J. Gen. Physiol..

[62] Clark, J. Exploring Ligand Binding in Glycine-Gated Ion Channels with Molecular Dynamics and Enhanced Sampling Methods. 2024 Ph.D. thesis; King’s College London: London, United Kingdom.

[63] Grudzinska J., Schemm R., Haeger S., Nicke A., Schmalzing G., Betz H., Laube B. (2005). The *β* subunit
determines the ligand binding properties of synaptic glycine receptors. Neuron.

[64] Dämgen M. A., Biggin P. C. (2020). A refined open state of the glycine
receptor obtained
via molecular dynamics simulations. Structure.

[65] Yu R., Hurdiss E., Greiner T., Lape R., Sivilotti L., Biggin P. C. (2014). Agonist and antagonist
binding in human glycine receptors. Biochemistry.

[66] Safar F., Hurdiss E., Erotocritou M., Greiner T., Lape R., Irvine M. W., Fang G., Jane D., Yu R., Dämgen M. A. (2017). The startle disease mutation E103K impairs
activation of human homomeric *α*1 glycine receptors
by disrupting an intersubunit salt bridge across the agonist binding
site. J. Biol. Chem..

[67] Spurny R., Ramerstorfer J., Price K., Brams M., Ernst M., Nury H., Verheij M., Legrand P., Bertrand D., Bertrand S. (2012). Pentameric ligand-gated ion channel ELIC is
activated by GABA and modulated by benzodiazepines. Proc. Natl. Acad. Sci. U. S. A..

[68] Hibbs R. E., Gouaux E. (2011). Principles of activation and permeation in an anion-selective
Cys-loop receptor. Nature.

[69] Kesters D., Thompson A. J., Brams M., Van Elk R., Spurny R., Geitmann M., Villalgordo J. M., Guskov A., Helena Danielson U., Lummis S. C. R., Smit A. B., Ulens C. (2013). Structural basis of
ligand recognition in receptors. EMBO Rep..

[70] Chung S.-K., Vanbellinghen J.-F., Mullins J. G. L., Robinson A., Hantke J., Hammond C. L., Gilbert D. F., Freilinger M., Ryan M., Kruer M. C., Masri A., Gurses C., Ferrie C., Harvey K., Shiang R., Christodoulou J., Andermann F., Andermann E., Thomas R. H., Harvey R. J., Lynch J. W., Rees M. I. (2010). Pathophysiological Mechanisms of
Dominant and Recessive GLRA1Mutations in Hyperekplexia. J. Neurosci..

[71] Paul F., Weikl T. R. (2016). How to Distinguish
Conformational Selection and Induced
Fit Based on Chemical Relaxation Rates. PLoS
Comput. Biol..

[72] Pless S. A., Lynch J. W. (2009). Magnitude of a conformational
change in the glycine
receptor *β*1-*β*2 loop
is correlated with agonist efficacy. J. Biol.
Chem..

[73] Calimet N., Simoes M., Changeux J.-P., Karplus M., Taly A., Cecchini M. (2013). A gating mechanism of pentameric ligand-gated ion channels. Proc. Natl. Acad. Sci. U. S. A..

