# Supplementary material for: Dynamics and Interplay of the Binding Pockets in the Glycine Receptor
Source: J Chem Inf Model. 2025 Jul 31;65(15):8194–206. doi: 10.1021/acs.jcim.5c00340 (PMC12344777; doi:10.1021/acs.jcim.5c00340)
Supplement: Supplementary file 1 [file ci5c00340_si_001.pdf]

Dynamics and Interplay of the Binding Pockets in the Glycine Receptor

Guangpeng Xue, Jacob Adam Clark, Cambrin Kemble-Diaz, Alessandro Crnjar, and Carla Molteni*

King's College London, Physics Department, Strand, London WC2R 2LS, UK

E-mail: carla.molteni@kcl.ac.uk

Supporting Information

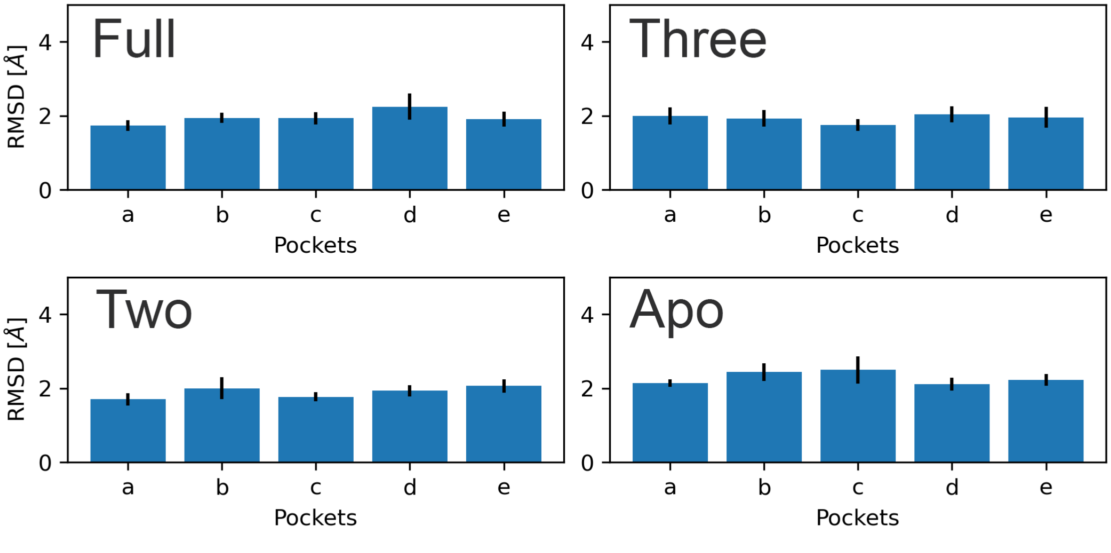

Figure S1: Root mean square deviation (RMSD) of the backbone atoms of the five ECD principal subunits (labeled as the corresponding binding pocket) of the GlyR models with different binding pocket occupancies calculated with respect to the initial structure of the production runs.

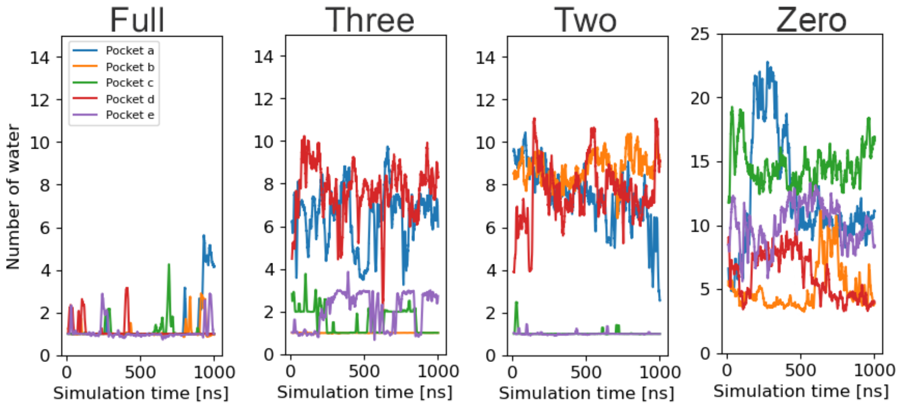

Figure S2: Time-evolution of the number of water molecules inside the pockets of the full-, three-, two-, and zero-ligand models.

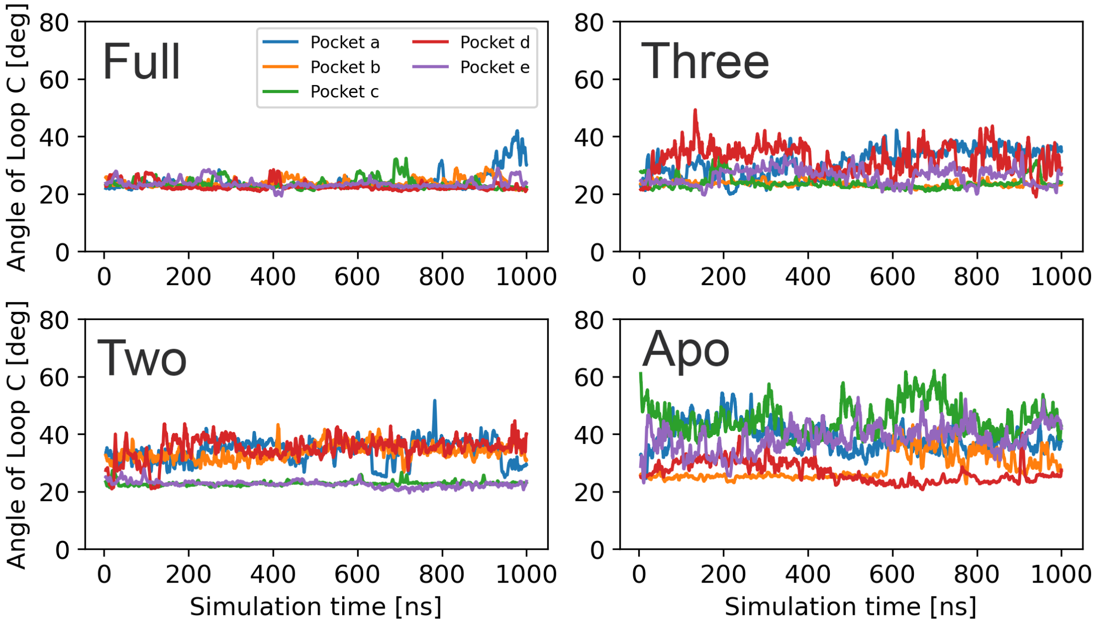

Figure S3: Time-evolution of the Loop C orientation angle of pockets of all models.

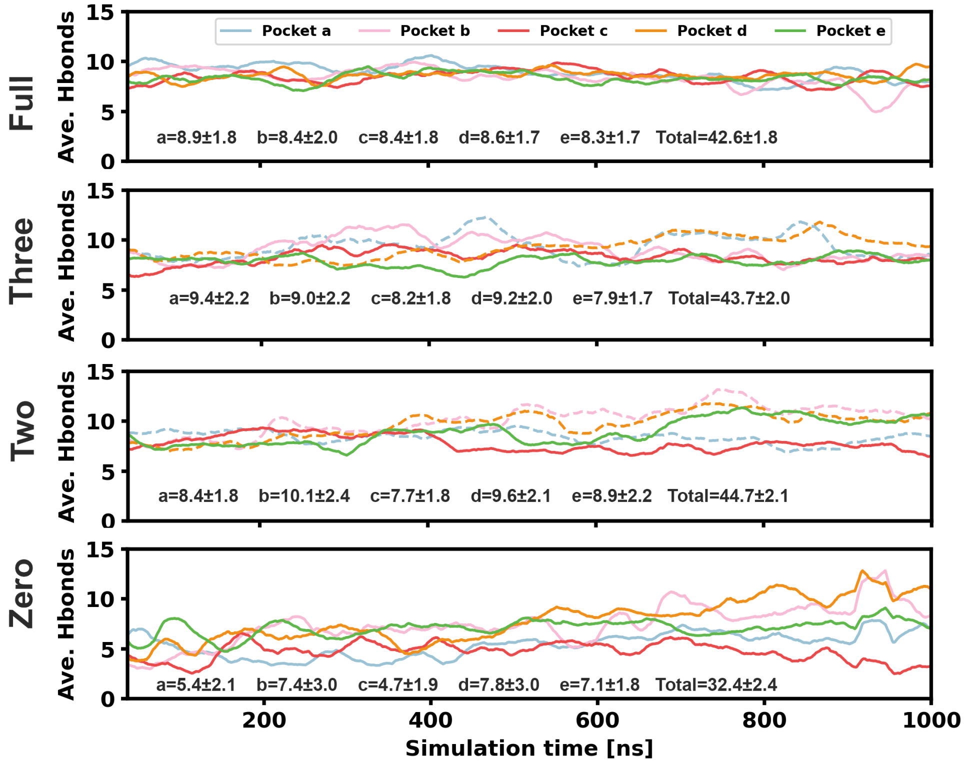

Figure S4: Time-evolution of the number of hydrogen bonds between adjacent subunits during the MD simulations. The average number of hydrogen bonds and their standard deviation during the MD simulations are shown. Solid lines refer to bound pockets, while dashed lines to empty pockets.

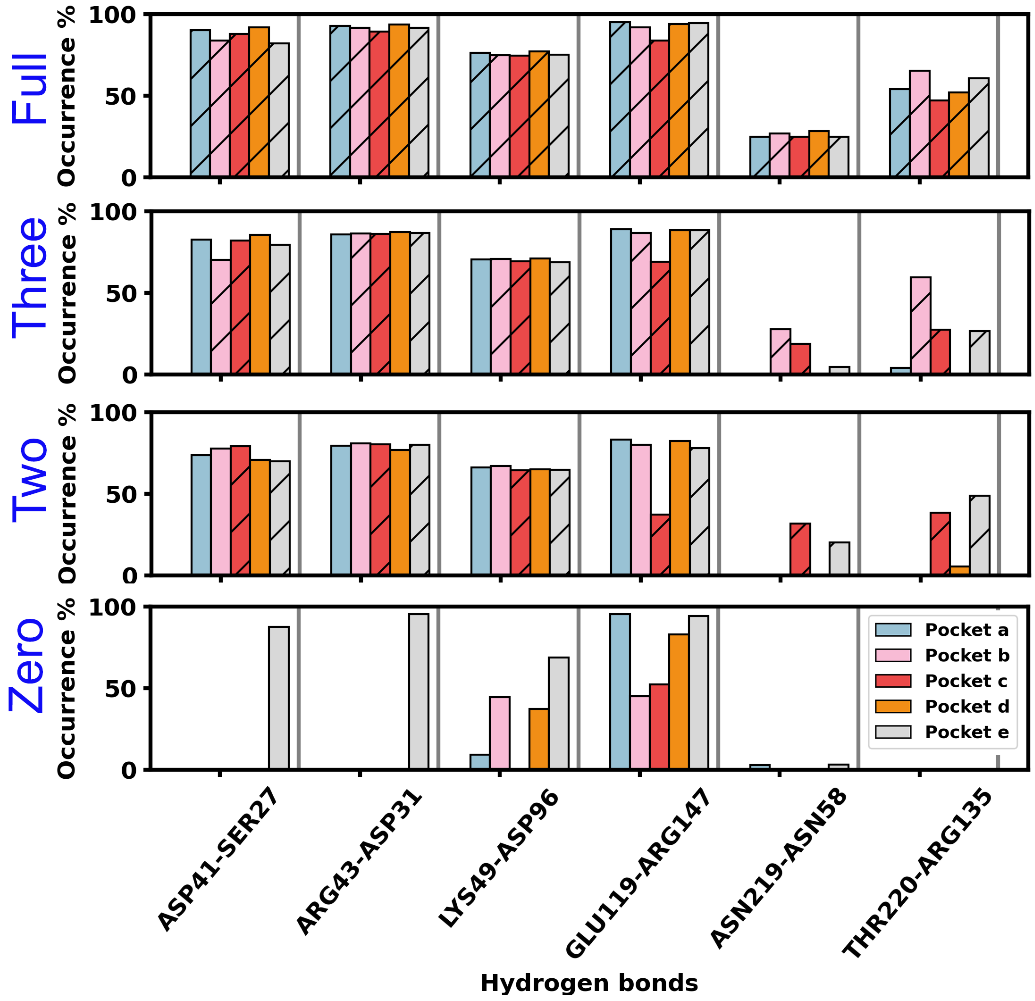

Figure S5: Hydrogen bond occurrence (in %) for selected pairs of residues at the interface between adjacent subunits. The first residue of the hydrogen bond is from the principal subunit and the second residue is form the complementary subunit. The histogram bars related to bound pockets are highlighted with slanted lines.

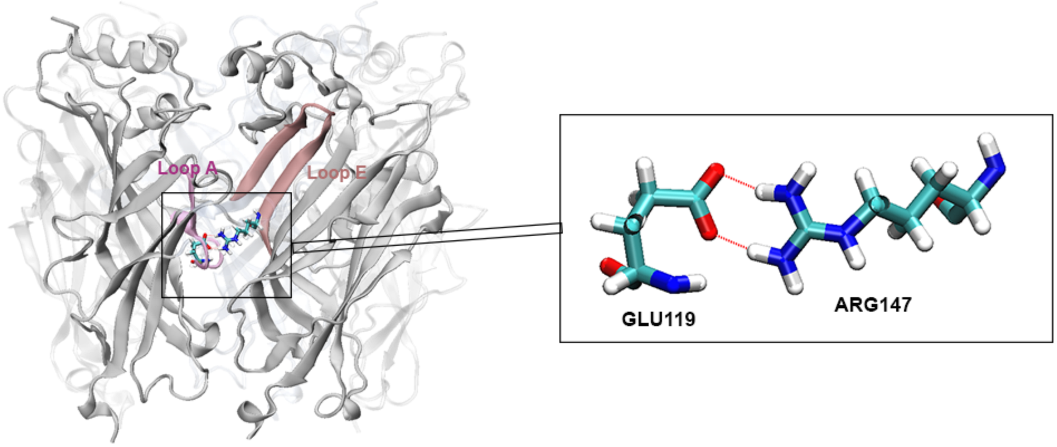

Figure S6: The salt-bridge between GLU119(+, Loop A) and ARG147(-, Loop E).

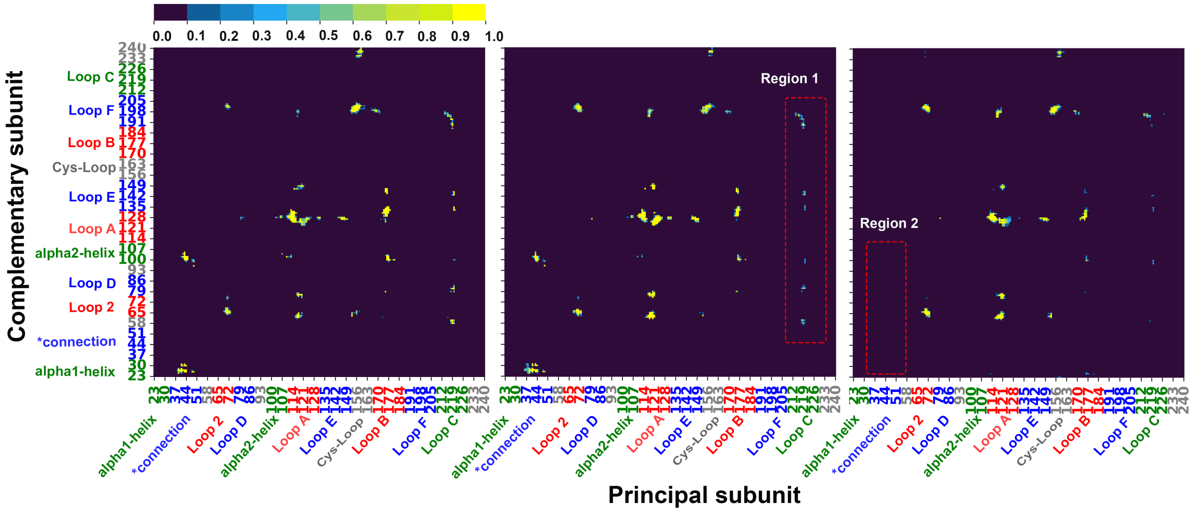

Figure S7: Selected protein distance contact map of adjacent subunits that form a binding pocket. On the left: the occupied pocket (b) of the three-ligand model; in the middle: the unoccupied pocket (a) of the three-ligand model (desensitized state); on the right the empty pocket (a) of the zero-ligand model (apo state). The colour-bar represents the fractional contact occurrence in the 1000 ns MD simulations. Residue labels are coloured according to the loop or secondary structure they belong to. "*connection" represents the loop connecting the alpha1-helix and beta1 loop. "Region 1" includes the distances between residues belonging to Loop C of principal subunit and residues of the complementary subunit. "Region 2" represents contacts in the N-terminus (top region) of the ECD model; the Apo structure has no contact here due to the tilting of subunits.

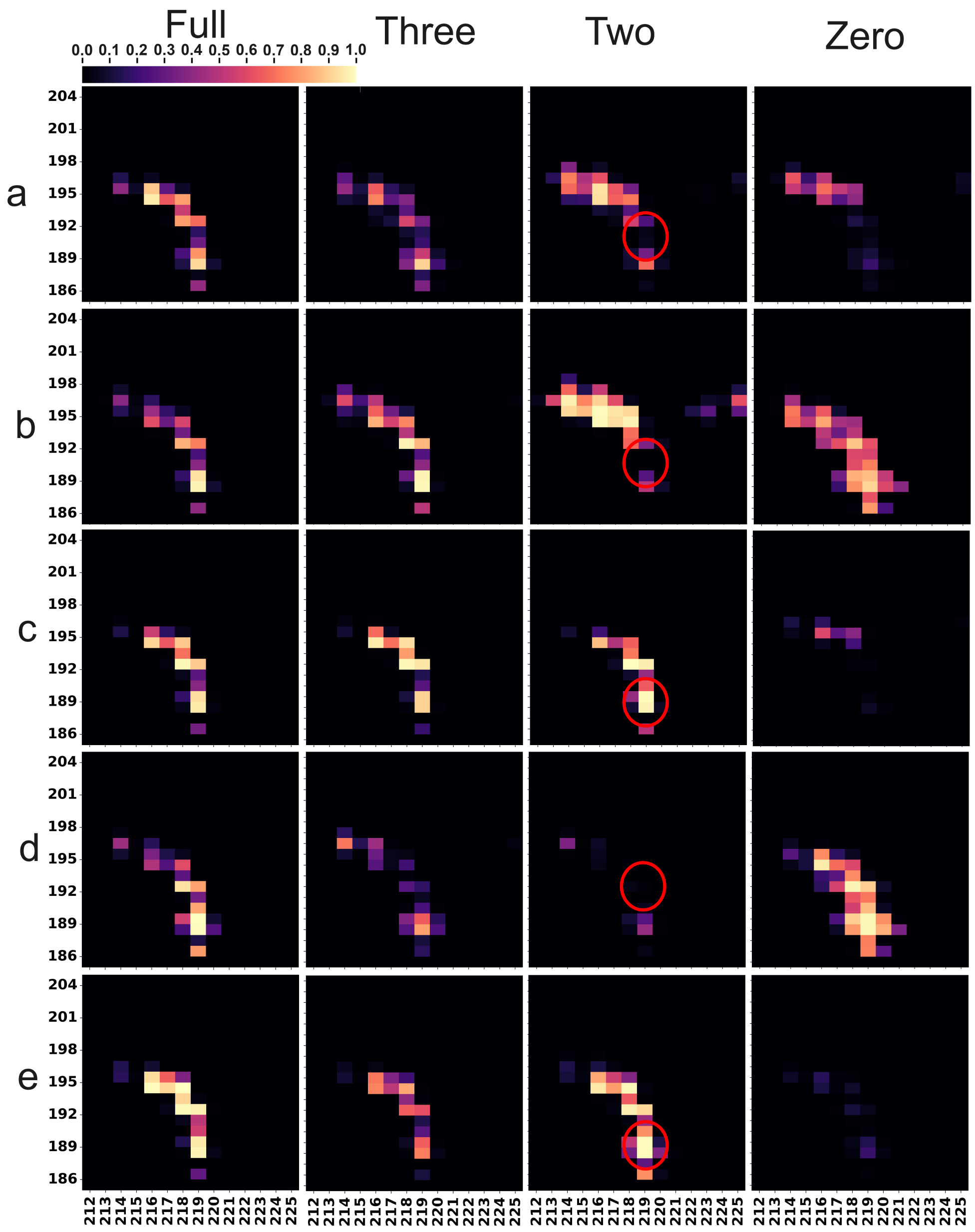

Figure S8: Zoomed-in protein distance contact map, focusing on the region of Loop C (x-axis, principal subunit) versus and Loop F (y-axis, complementary subunit).

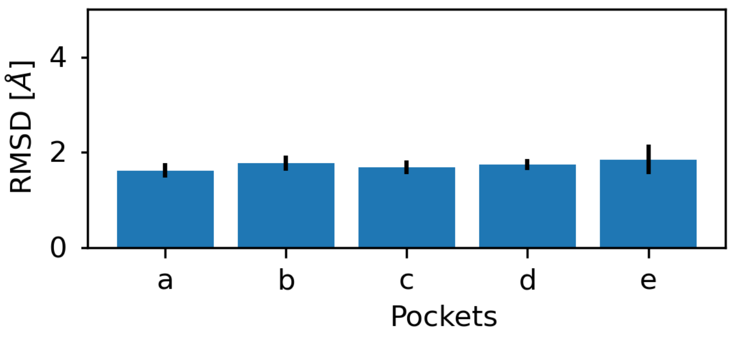

Figure S9: Two-ligand model (consecutive occupied pockets (d) and (e)). Root mean square deviation of the backbone atoms of the five ECD principal subunits of the GlyR model, calculated with respect to the initial structure of the production runs.

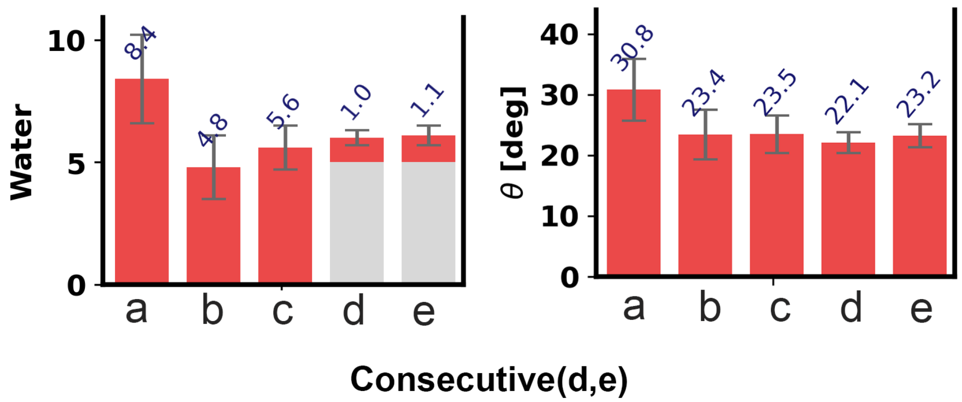

Figure S10: Two-ligand model (consecutive occupied pockets (d) and (e)). On the left: the average number of water molecules in both occupied and unoccupied binding pockets, with values for occupied pockets raised by 5 to somehow account for the presence of glycine and make it visually comparable to the unoccupied pocket content; on the right: the average angle (in degrees) representing the orientation of Loop C across all pockets.

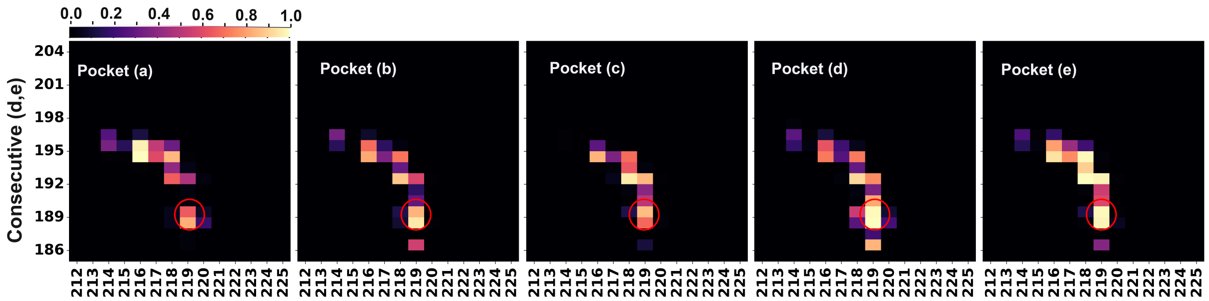

Figure S11: Two-ligand model (consecutive occupied pockets (d) and (e)). Protein distance contact map, focusing on the residues of Loop C (X-axis, principal subunit) versus those of Loop F (Y-axis, complementary subunit).

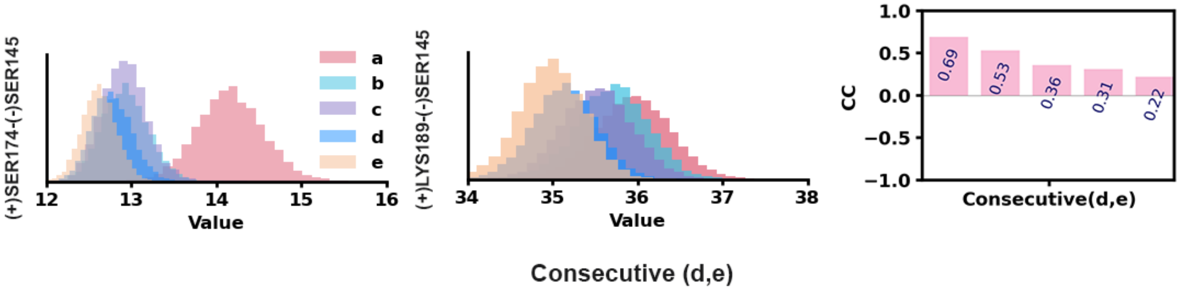

Figure S12: Two-ligand model (consecutive occupied pockets (d) and (e)). On the left: histograms of the distance between SER174 (+, Loop B′) and SER145 (-, Loop E″); in the middle: distance between LYS189 (+, Loop F′) and SER145 (-, Loop E″); on the right: correlation coefficient between the two distances for the five pockets.

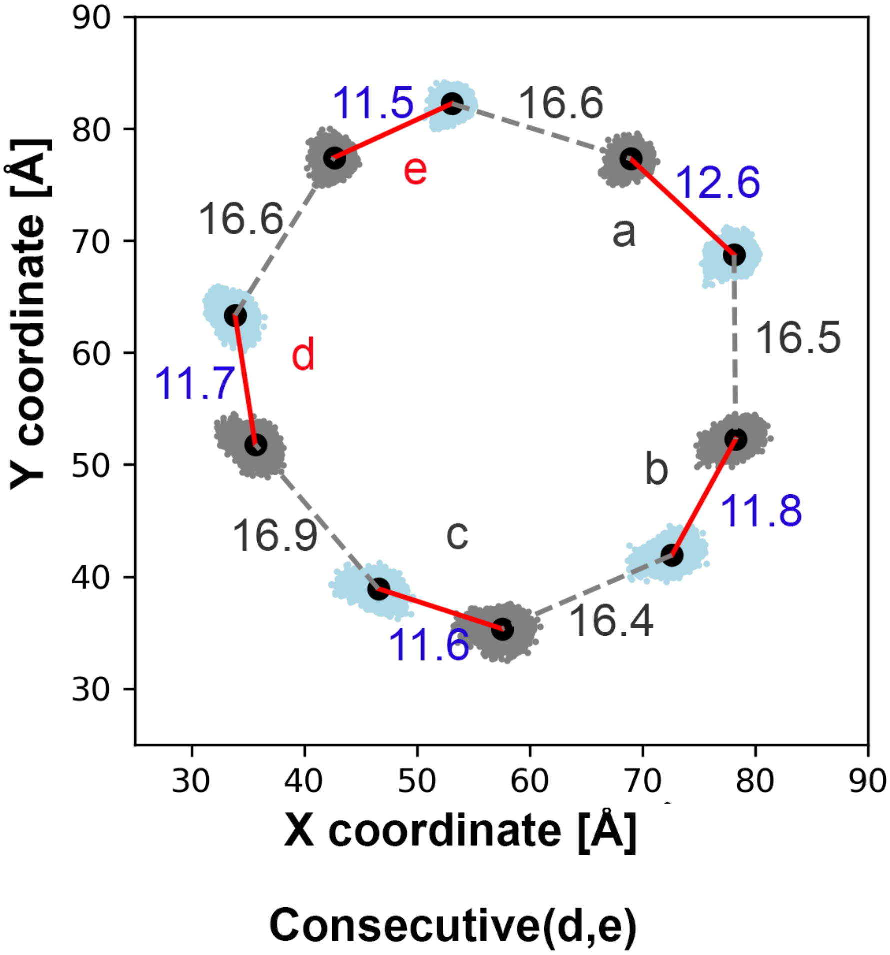

Figure S13: Two-ligand model (consecutive occupied pockets (d) and (e)). The positions of the centers of mass of the binding pockets in the plane orthogonal to the channel axis are shown in the X-Y plane. The pockets are labelled (a) to (e) for each model. For each pocket the two scatter distributions represent the centers of mass of the C_α of the binding residues respectively on the principal (light blue) and on the complementary (gray) subunits that form the pocket. The labels of unbound pockets are in gray, while those of bound pockets are in red. Gray dash lines connect scatter distributions in the same subunit indicating inter-pocket distances (values in Å shown in gray), while red solid lines represent intra-pocket distances (values in Å shown in blue).

Table S1: Intra-pocket (a, b, c, d and e) and inter-pocket (a-b, b-c, c-d, d-e and e-a) distances (in Å) in the two-ligand models. Intra-pocket distances of bound pockets are in boldface. ⟨ and ⟩ indicate participation to the bound pocket "on the left" and/or "on the right".

Occupied pockets	a	a-b	b	b-c	c	c-d	d	d-e	e	e-a
c,e	12.8	15.8	12.6	16.4⟩	**11.7**	⟨16.5	12.4	16.5⟩	**11.4**	⟨16.5
d,e	12.6	16.5	11.8	16.4	11.6	16.9⟩	**11.7**	⟨16.6⟩	**11.5**	⟨16.6
